# A hybrid pelican-GWO optimized fractional order PID controller for enhanced performance of hybrid active power filters

**DOI:** 10.1038/s41598-026-45958-4

**Published:** 2026-04-15

**Authors:** Rehab S. Salah Eldeen, Ahmed Deyaa Elkoshairy, Hala M. Abdel Mageed, Adel A. E. M. S. Ahmed

**Affiliations:** 1 National Institute of Standards (NIS), Giza, Egypt; 2 Electrical Engineering Dept, Faculty of Engineering, Ain Shams University, Cairo, Egypt

**Keywords:** POA-Pelican Optimization Algorithm, FOPID Fractional Order PID (FOPID) Controller, GWO Grey Wolf Optimizer, Hybrid Active Power Filter (HAPF), Energy science and technology, Engineering, Mathematics and computing

## Abstract

Hybrid Active Power Filter (HAPF) performance is strongly affected by the nonlinear behavior and tight coupling of control parameters, which makes traditional optimization techniques prone to unstable tuning and unreliable performance when applied to fractional-order controllers. This paper proposes an advanced control framework for HAPFs based on a novel hybrid meta-heuristic optimization approach. The method combines the adaptive search capability of the Pelican Optimization Algorithm (POA) with the social intelligence of the Grey Wolf Optimizer (GWO) to achieve a more balanced and reliable tuning process than standalone methods to efficiently tune all five parameters of a Fractional Order PID (FOPID) controller. The objective is to improve dynamic stability and harmonic attenuation under diverse operating conditions. Simulations carried out in the MATLAB/Simulink (R2018a) environment demonstrate that the proposed hybrid POA-GWO approach outperforms conventional PID controllers and FOPID controllers optimized using single algorithms. Key improvements include significant reduction in total harmonic distortion (THD) where THD of source current reduces from 28.95% to 4.34%, also the proposed hybrid FOPID controller demonstrates faster convergence and achieves a lower objective function value compared to individual optimization algorithms and conventional controllers, The results also demonstrate enhanced durability under balanced and unbalanced loading conditions. The results confirm the effectiveness of the proposed controller as a practical solution for real-time power quality enhancement in emerging smart grid applications.

## Introduction

 The increasing integration of nonlinear and time-varying loads into modern electrical networks has significantly heightened concerns related to power quality. One of the most prominent manifestations of these issues is the distortion of voltage and current waveforms due to harmonics. These disturbances can severely impact the stability, efficiency, and reliability of power systems. With the rapid expansion of smart grid technologies and applications, addressing power quality problems has become even more critical to ensure the optimal performance of modern electrical infrastructures^1^.

Hybrid Active Power Filters (HAPFs) have become a popular solution for mitigating harmonics in power systems, as they combine the advantages of both passive and active filtering techniques. Traditional passive filters, which rely on inductors and capacitors, have been widely used to reduce harmonic distortion and compensate reactive power^2^. For example, studies have shown that passive filters can be effectively designed for practical applications, from laboratory setups^3^ to industrial environments like steel mills^4^. Active power filters, in contrast, use power electronic converters and advanced control strategies to dynamically cancel harmonic currents. These approaches offer real time compensation and improved flexibility compared to passive filters. Research has demonstrated various active control techniques, including synchronous reference frame methods^5^, simulation based design using MATLAB^6^, and comparative analyses of different controller performances^7^. Reviews of optimal filter placement and sizing further highlight the importance of design optimization for maximum efficiency^8^. By combining passive and active elements, HAPFs provide a more robust and adaptable solution, achieving better harmonic mitigation and power quality improvement than either method alone. HAPFs combine the advantages of both and provide better performance in harmonic reduction and reactive power compensation. The performance of a Hybrid Active Power Filter (HAPF) is largely governed by the effectiveness of its control strategy.

Over the past decade, several enhanced PID-based structures have been proposed to address the inherent limitations of classical PID controllers, particularly in systems affected by uncertainties and disturbances. One notable advancement is the robust formulation of the fractional-order PID (FOPID) controller, where robustness-oriented tuning strategies are employed to enhance stability margins and sensitivity characteristics under parameter variations. These approaches aim to exploit the flexibility of fractional calculus while maintaining reliable performance in practical control systems^9,10^.

Another significant development is the three-degree-of-freedom (3-DoF) PID controller. Unlike the conventional PID structure, the 3-DoF formulation allows independent tuning of the reference tracking and disturbance rejection paths. This structural flexibility enables improved transient performance without significantly compromising robustness, making it attractive for industrial and power electronic applications^11^^,^^12^.

The PIDA (Proportional–Integral–Derivative–Acceleration) controller further extends the conventional PID structure by incorporating an acceleration term to improve high-frequency compensation and response speed. Similarly, the TID (Tilt–Integral–Derivative) controller replaces the proportional term with a fractional tilt component, enhancing low-frequency behavior and robustness while preserving structural simplicity^13,14^.

While traditional Proportional Integral Derivative (PID) controllers are widely adopted due to their simplicity and ease of implementation, they often fall short in addressing the complex dynamic behavior and inherent nonlinearities of real-world power systems. In contrast, the Fractional Order PID (FOPID) controller an extension of the classical PID introduces two additional tuning parameters, integral order (µ) and derivative order (λ). These fractional orders enhance the controller’s tuning flexibility and offer superior dynamic response and control precision, making FOPID a more robust and adaptable solution for modern power quality applications^15^.

In general, conventional PID controllers exhibit limited effectiveness when applied to nonlinear, uncertain, and strongly coupled systems. Despite their widespread use and several inherent advantages such as simplicity and ease of implementation their performance often lacks sufficient robustness and disturbance rejection capability, particularly in complex and dynamically changing environments^16^. One of the main challenges of implementing FOPID controllers is the complexity of tuning, as it requires optimizing five parameters (Kp, Ki, Kd, λ, µ)^16^. Fractional-order control introduces extra flexibility in system design, allowing more precise shaping of dynamic responses compared to conventional PID controllers. Research has shown that fractional order systems offer better modeling capabilities and enhanced robustness^18^^,^^19^. They provide clear advantages in the frequency domain, such as improved stability and more accurate phase margin adjustment^20^. Comparisons between classical PID and FOPID controllers indicate that FOPID achieves smoother responses and greater resilience to parameter variations and external disturbances, especially when optimized using intelligent algorithms^21^. While traditional PID methods can handle time-delay systems reasonably well, fractional-order controllers give designers additional tuning freedom and improved overall performance^22^. In short, the fractional nature of FOPID controllers makes them particularly suitable for applications requiring precise stability, refined transient and steady-state behavior, and more flexible response control.

### literature review

Hybrid Active Power Filters (HAPFs) have gained significant attention as an efficient way to address power quality issues in systems with nonlinear loads. To improve their control accuracy and dynamic response, many recent works have focused on optimizing the tuning of controller parameters. Different optimization techniques have been applied to both conventional PID and fractional order PID (FOPID) controllers to achieve lower harmonic distortion and better reactive power compensation. The following section reviews selected studies that employ various optimization methods in HAPF applications, highlighting their algorithms, performance, and limitations. Recently, various nature-inspired and bio-inspired optimization algorithms have been widely applied in power and energy systems to enhance control performance and dynamic stability. For instance, Mukherjee and Banerjee (2025) employed the Symbiotic Organism Search (SOS) algorithm to optimize a STATCOM-based control scheme in a hybrid wind–diesel power system^23^. Another notable example of advanced metaheuristic application in power systems is the work by Khadanga et al. (2023), which introduced a sine augmented scaled arithmetic optimization algorithm for frequency regulation in a microgrid with virtual inertia control^24^. Also, Fathollahi-Fard et al. proposed the Red Deer Algorithm (RDA)^25^, Social Engineering Optimizer (SEO)^26^, which demonstrated promising results in solving complex, nonlinear, and multi-objective optimization problems in engineering applications. These studies highlight the growing interest in employing nature-inspired and bio-inspired optimization techniques due to their strong global search capability and robustness in handling highly nonlinear power system models. Thuyen (2019) proposed a design approach for Hybrid Active Power Filters using the Social Spider Algorithm (SSA) to reduce total harmonic distortion and improve reactive power compensation. The method showed good simulation results in harmonic mitigation, but it lacked experimental validation and had relatively high computational demand^27^. Nagaraju and Chandramouli (2023)introduced a Pigeon-Inspired Optimization (PDO) to fine tune FOPID controller for a Unified Power Quality Conditioner in grid-connected hybrid renewable systems to reduce harmonic distortion and improve voltage stability. The controller tuned FOPID parameters using Proportional Derivative Optimization and achieved better THD reduction in simulations, but no experimental validation was provided^28^. Mishra et al. (2020) developed a FOPID based Hybrid Shunt Active Power Filter optimized using the PSO-GWO algorithm to reduce THD and enhance reactive power compensation. The method achieved better harmonic mitigation and voltage regulation, but the computational complexity of the hybrid optimization increased the processing time^28^. Ahmed et al. (2021)proposed a fractional order PID controller for a Permanent Magnet Synchronous Motor (PMSM) drive, optimized using a hybrid Grey Wolf Optimization algorithm to enhance dynamic response and minimize speed error. The study demonstrated superior performance in terms of reduced overshoot, faster settling time, and lower steady state error compared to conventional PID controllers. However, the approach was validated only through simulations and involved increased computational effort due to the hybrid optimization process^30^. Alasali et al. (2022) proposed optimal control strategies for Hybrid Active Power Filters using the Whale Optimization Algorithm (WOA) to minimize harmonic distortion and improve power quality. The approach achieved significant THD reduction and better transient response in simulations. However, the study was limited to simulation analysis without experimental validation or hardware implementation^30^. Pandey et al. (2023) developed a Rabbit Optimization Algorithm (ROA) based tuning method for a Fractional Order PI controlled Hybrid Active Power Filter aimed at enhancing power quality. The study focused on minimizing Total Harmonic Distortion (THD) and improving reactive power compensation under nonlinear load conditions. The proposed ROA-FOPI controller outperformed conventional and other optimized controllers in terms of THD reduction and dynamic performance. However, the research was limited to simulation results, lacking experimental verification^32^. Ali et al. (2023)developed a Hybrid Marine Predator Sine Cosine Algorithm (HMPSCA) to tune the parameters of a Hybrid Active Power Filter (HAPF) aiming to enhance power quality. The approach efficiently minimized THD and improved reactive power compensation through a balance between exploration and convergence speed. Simulation outcomes confirmed its effectiveness compared to conventional optimization methods, though it lacked hardware implementation and involved high computational effort^33^. Another relevant study by Idir et al. (2025) investigated metaheuristic optimization for a fractionalized‑order PID controller in a vehicle cruise control system. The research compared classical PID tuning with fractionalized‑order PID tuned using Harris Hawks Optimization (HHO), Genetic Algorithm (GA), and Particle Swarm Optimization (PSO). Simulation results demonstrated that the fractionalized controller significantly outperformed the conventional PID in terms of transient response and robustness, with the HHO‑based design providing the best overall balance between responsiveness and stability. This work highlights the effectiveness of combining fractional control structures with metaheuristic optimization methods across different engineering applications^33^. Aldosary (2024) proposed a Fractional-Order PID (FOPID) controller optimized through a hybrid Jellyfish Search–Particle Swarm Optimization (JF-PSO) algorithm to enhance power quality conditioners. The method focused on reducing total harmonic distortion (THD) and improving dynamic response under varying load conditions. Simulation results showed superior performance compared to classical controllers however, the study was limited to simulations, and the hybrid optimization increased computational complexity^35^. In the context of fractional controller development beyond conventional FOPID designs, Guedida et al. (2024) proposed a reduced‑order fractionalized proportional–integral controller (ROFPI) for disturbance compensation in a dual‑star induction motor drive based on direct torque control. The study employed a modified switching table to eliminate circulation currents and achieve nearly sinusoidal current forms, and comparative simulations showed that the fractionalized controller provided superior transient speed and torque responses with lower overshoot and faster settling than conventional controllers under various operating conditions. Such research highlights alternative fractional control structures and further supports the utility of fractionalized control strategies in improving dynamic performance across different engineering systems^36^. In addition to the optimization strategies discussed earlier, recent conference research has explored the use of nature‑inspired metaheuristic algorithms such as the Marine Predators Algorithm (MPA) for controller tuning in microgrid applications. Balabantaraya et al. (2024) applied MPA to improve frequency stability in a maritime microgrid using a fractional‑order PID controller, demonstrating enhanced dynamic performance under varying operating conditions. This further highlights the expanding scope of metaheuristic methods in power system control and motivates the use of hybrid optimization techniques for advanced controller tuning tasks^37^. Another recent study by Mishra et al. (2024) introduced an improved equilibrium optimization (i‑EOA) to fine‑tune a fuzzy tilted double integral derivative with filter (F‑TIDF‑2) controller for frequency regulation of an off‑grid microgrid. The enhanced equilibrium optimization algorithm addresses typical metaheuristic challenges such as local optima trapping and population diversity, and the optimized controller demonstrated superior frequency stabilization and dynamic performance compared with conventional control schemes under stochastic load and generation variations. This work further illustrates the expanding use of advanced metaheuristic methods in power system control optimization and supports the exploration of hybrid strategies for fractional‑order controller tuning^38^. Herman et al. (2024) presented a hybrid control approach for a Hybrid Active Power Filter (HAPF) combining proportional–resonant (PR) and predictive control techniques to enhance harmonic suppression and reactive power compensation in distribution systems. The study analyzed the controller’s effectiveness using THD, dynamic response, and power factor as performance metrics. Results showed effective harmonic reduction, but the approach involved high computational complexity and was only validated in simulations, without experimental verification. [39] Guven and Mengi (2024) explored the use of nature-inspired optimization algorithms to tune FOPID controllers for time delayed control systems. The main goal was to enhance system stability and transient response by optimally adjusting FOPID parameters using algorithms such as Genetic Algorithm (GA), Particle Swarm Optimization (PSO), and Grey Wolf Optimizer (GWO). The study evaluated performance using time domain metrics like rise time, overshoot, and Integral Time Absolute Error (ITAE). Simulation results showed improved performance compared to traditional PID controllers. However, the approach was limited to simulation validation and involved high computational cost due to the complex search process of metaheuristic algorithms^39^. Nanda et al. (2025) proposed a hybrid shunt active power filter (HSAPF) controlled by a Kalman Filter–based sliding mode adaptive fuzzy PID tuned using the Teaching-Learning Optimization (TLO) algorithm to enhance power quality. The study aimed to minimize harmonic distortion and improve reactive power compensation under varying load conditions. Performance was evaluated mainly through THD reduction and dynamic response, showing improved results compared to conventional methods. However, the work was limited to simulation analysis without hardware validation, and the algorithm’s computational complexity may restrict real-time implementation^41^. Although several studies have applied metaheuristic optimization to tune fractional-order PID controllers in power systems, many face limitations such as slow convergence, suboptimal harmonic mitigation, and reduced robustness under unbalanced load conditions. These shortcomings motivate the development of the proposed hybrid Pelican–GWO approach, which aims to achieve faster convergence, lower THD, and improved performance under diverse operating scenarios.

### Robustness of fractional-order PID Controllers under uncertainties and disturbances

Recent research has consistently shown that fractional-order PID (FOPID) controllers tuned with optimization techniques provide enhanced robustness against uncertainties and disturbances compared to traditional integer-order PID schemes. For instance, in power control of a nuclear reactor model, an optimized FOPID controller demonstrated superior disturbance rejection and smaller tracking errors under model uncertainties and external perturbations, outperforming the classic PID approach in terms of transient response and robustness in setpoint tracking^42^. Similarly, advanced FOPID-based designs have been applied to renewable energy and load frequency control problems, where they maintained performance under variations in load demand and noisy operating conditions, indicating improved disturbance accommodation and robustness to parameter changes^43^. Moreover, fuzzy logic enhancements to fractional controllers have been shown to further strengthen dynamical resilience in power electronic systems (such as DC-DC converters), exhibiting stable performance with variable load and component deviations^44^. These collective findings support the idea that optimized and hybrid FOPID controllers can effectively cope with different patterns of uncertainties and disturbances, which justifies their use for validating the efficacy of the proposed tuning algorithms in this work.

This study introduces a novel hybrid optimization approach (hybrid POA–GWO) that merges the exploration strength of the Pelican Optimization Algorithm (POA) with the adaptive hunting strategy of the Grey Wolf Optimizer (GWO) to achieve precise and efficient parameter tuning of the Fractional Order PID (FOPID) controller. By exploiting the complementary characteristics of these two algorithms POA’s strong global search ability and GWO’s effective local exploitation the proposed framework ensures an improved balance between convergence speed and solution accuracy. This makes the controller design more robust and adaptive to the complex, nonlinear behavior of modern power systems.

The hybrid approach leverages the strong exploration ability of POA and the adaptive convergence of GWO to achieve precise and efficient parameter optimization. The controller is developed and tested in MATLAB/Simulink under both balanced and unbalanced loading scenarios. Simulation results demonstrate that the proposed hybrid FOPID controller achieves significant improvements in harmonic reduction, dynamic response, and system robustness compared with conventional PID and single-algorithm-based FOPID controllers.

### Research gaps and motivation

Although various optimization algorithms have been applied to tune FOPID controllers in hybrid active power filters, most studies rely on single algorithm strategies. These approaches often face limitations such as slow convergence, premature stagnation, or reduced accuracy when dealing with complex nonlinear systems. In addition, the performance of conventional PID controllers remains insufficient in ensuring both harmonic suppression and robustness under different load conditions. However, the Pelican Optimization Algorithm (POA), a recently developed metaheuristic, has not yet been explored in HAPF control applications. A detailed literature review confirms that no previous research has investigated the integration of POA or a hybrid Pelican-GWO framework for optimizing FOPID or PID parameters in HAPFs. Therefore, this study introduces a novel hybrid Pelican-GWO optimization technique to enhance the controller performance and achieve superior power quality improvement. Hence, this study represents an initial attempt to investigate the applicability of the Pelican Optimization algorithm and hybrid POA-GWO in controlling HAPFs based on FOPID controller, laying the groundwork for future comparisons with other optimization techniques. The proposed hybrid POA–GWO approach uses a sequential combination of the Pelican Optimization Algorithm (POA) and the Grey Wolf Optimizer (GWO), making it different from earlier hybrid methods that often run algorithms in parallel or switch randomly between them. In this approach, POA is first employed to explore the search space broadly and identify promising solutions, avoiding early stagnation. Then, GWO is applied to refine these solutions through focused local search. By letting each algorithm operate in the phase it performs best, this strategy improves the stability of convergence and the reliability of FOPID tuning. Unlike previous hybrids, this method does not require extra control parameters and shows consistent performance even under varying load conditions, making it especially suitable for HAPF control applications.

**In summary**,** the key contributions of this study are**:

1. Introduction of a novel sequential hybrid Pelican-GWO optimization strategy for FOPID controller tuning in HAPFs, effectively combining global exploration and local exploitation.
2. Demonstration of improved convergence speed, reduced total harmonic distortion (THD), and enhanced robustness under both balanced and unbalanced load conditions compared to conventional PID/FOPID controllers and single-algorithm optimizations.
3. Provision of a practical and parameter efficient framework suitable for real-time HAPF applications, laying the groundwork for future comparative studies with other metaheuristic techniques.

### Paper organization

The paper is organized as follows. Section 1 introduces the study and includes background, literature review, the identified research gap with motivation, and the structure of the paper. Section 2 presents the modeling of the hybrid active power filter, while Sect. 3 explains the Optimization techniques applied in this work covering fraction order PID controller, Pelican Optimization and Grey Wolf Optimization. Section 4 reports the results and analysis, covering passive filter results, hybrid filter optimized by hybrid POA-GWO techniques, results under unbalanced conditions and comparing between optimizations. Finally, Sect. 5 summarizes the main conclusions of the study.

## Modeling of hybrid active power filter

**Fig. 1:**
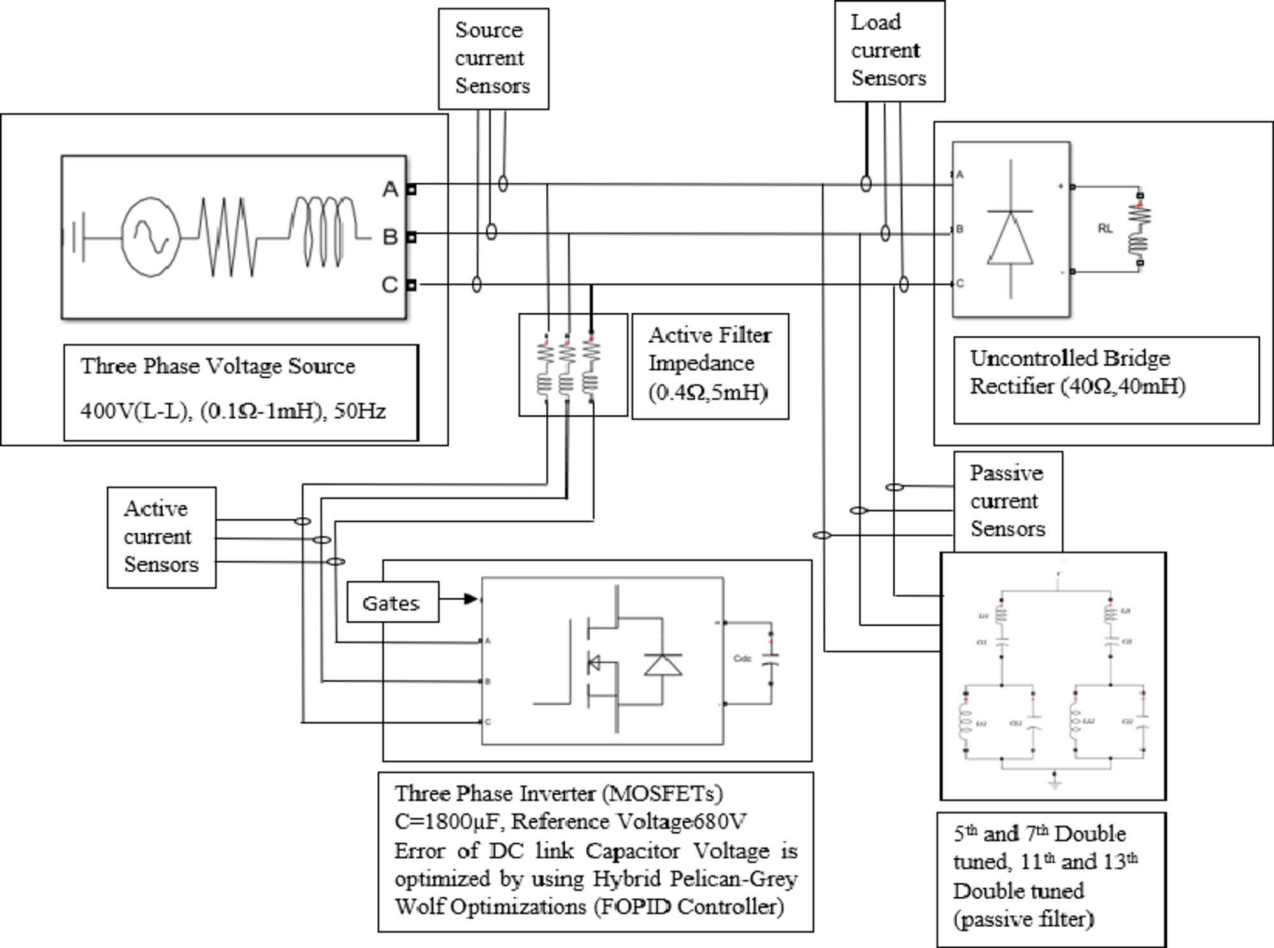
Hybrid Active Power Filter Block Diagram.

A simulation model of a Hybrid Active Power Filter has been developed using MATLAB/Simulink. The setup comprises a three-phase voltage source connected to a three-phase rectifier, which supplies an RL load that acts as a source of harmonic distortion. Both passive and active filters are incorporated between the source and the load, as illustrated in Fig. 1.

The passive filtering stage includes two double-tuned filters designed specifically to mitigate the 5th and 7th, as well as the 11th and 13th harmonic components. The remaining harmonic distortions are addressed by the active power filter.

Current sensors are deployed to measure the source and load currents, while voltage sensors monitor the source voltage. These measurements provide essential input signals for the active filter’s control system.

The control circuit constitutes the core of the HAPF, functioning as the central processing unit responsible for harmonic analysis, compensating current estimation, and the generation of switching signals to control the MOSFETs within the voltage source inverter (VSI).

This study employs the instantaneous power theory (p–q theory) as the foundation for the control strategy. The instantaneous power (P-Q) theory is employed to generate the reference current for the shunt active filter. This approach allows rapid identification of the harmonic and reactive components of the distorted load current through the α–β transformation. Unlike the synchronous reference frame (d–q) method, the P–Q theory is simpler to implement since it eliminates the need for a phase-locked loop, enhancing its suitability for real-time applications. Moreover, it ensures stable and efficient performance under both steady-state and dynamic load conditions, making it a practical choice for the proposed hybrid active power filter system^45,46^.

**Fig. 2:**
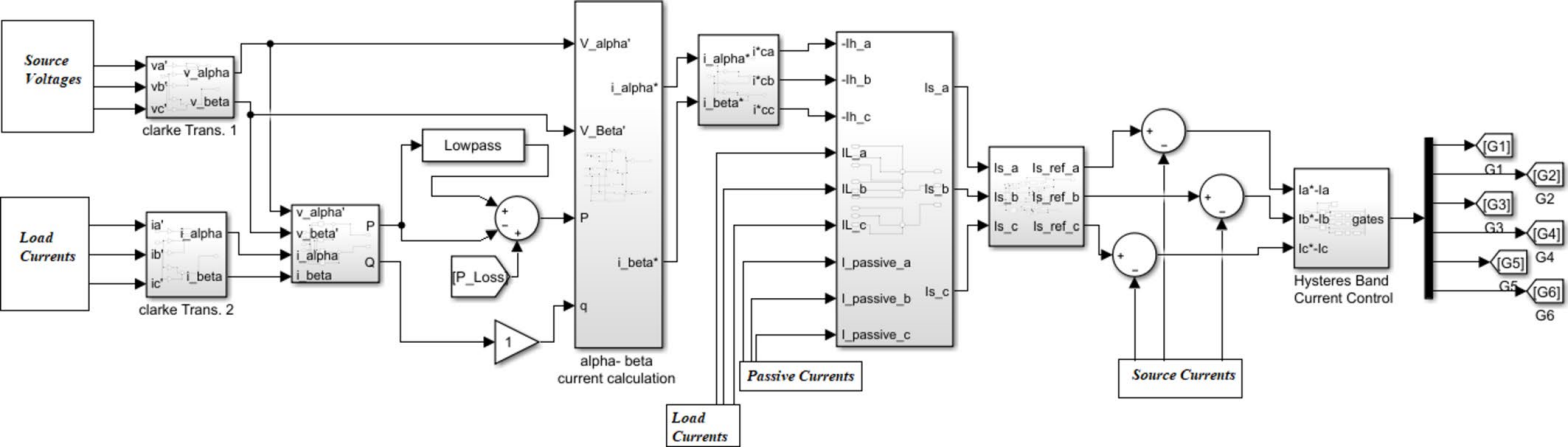
Control process to obtain source reference currents using MATLAB/Simulink.

Initially, the three-phase voltage and current signals are transformed into two orthogonal components (α, β) through the Clarke transformation. The d-axis component of the current is passed through a low-pass filter to extract the fundamental frequency component, and the harmonic current is derived by subtracting this filtered fundamental from the original signal. Simultaneously, the q-axis component primarily contains the harmonic components. Subsequently, the inverse Clarke transformation is applied to convert the (α, β) components back into three-phase reference currents. These reference currents are the input to a hysteresis current controller, which generates the gating pulses necessary to drive the MOSFETs of the VSI. The overall control scheme is depicted in Fig. 2.

Equations (1), (2), and (3) express the instantaneous voltages of phases a, b, and c, respectively, as sinusoidal functions of time, where V_max_ represents the peak voltage and ωt denotes the instantaneous phase angle.

1 $$\:Va={V}_{max}\mathrm{s}\mathrm{i}\mathrm{n}\left(wt\right)$$

2 $$\:Vb={V}_{max}\mathrm{s}\mathrm{i}\mathrm{n}(wt-120^\circ)$$

3 $$\:Vc={V}_{max}\mathrm{s}\mathrm{i}\mathrm{n}(wt+120^\circ)$$

Through the application of the Clarke transformation, the three-phase load quantities are transformed into two orthogonal components, namely α and β.

4 $$\:\left[\begin{array}{c}v\alpha\:\\\:v\beta\:\end{array}\right]=\sqrt{\frac{2}{3}}\left[\begin{array}{ccc}1&\:\frac{-1}{\sqrt{2}}&\:\frac{-1}{\sqrt{2}}\\\:0&\:\frac{\sqrt{3}}{2}&\:\frac{-\sqrt{3}}{2}\end{array}\right]\left[\begin{array}{c}va\\\:vb\\\:vc\end{array}\right]$$

5 $$\:\left[\begin{array}{c}iL\alpha\:\\\:iL\beta\:\end{array}\right]=\sqrt{\frac{2}{3}}\left[\begin{array}{ccc}1&\:\frac{-1}{\sqrt{2}}&\:\frac{-1}{\sqrt{2}}\\\:0&\:\frac{\sqrt{3}}{2}&\:\frac{-\sqrt{3}}{2}\end{array}\right]\left[\begin{array}{c}iLa\\\:iLb\\\:iLc\end{array}\right]$$

Equations (6) and (7) are utilized to calculate the instantaneous active and reactive power.

6 $$\:P=v\alpha\:.i\alpha\:+v\beta\:.i\beta\:$$

7 $$\:q=v\beta\:.i\alpha\:-v\alpha\:.i\beta\:$$

To isolate the harmonic power, a low-pass filter is initially used to extract the fundamental power component, which is subsequently subtracted from the total (net) power.

8 $$\:{P}_{har.}=P-{P}_{Fund.}$$

The harmonic power is combined with the loss power associated with the inverter’s DC-link capacitor voltage. Accordingly, the harmonic current component (− I harmonics) is determined using the following Eq. 

9 $$\:\left[\begin{array}{c}i\alpha\:\\\:i\beta\:\end{array}\right]=\frac{1}{{v}_{\alpha\:}^{2}+{v}_{\beta\:}^{2}}\left[\begin{array}{cc}v\alpha\:&\:v\beta\:\\\:v\beta\:&\:-v\alpha\:\end{array}\right]\left[\begin{array}{c}{P}_{loss}+P-{P}_{Fund.}\\\:-q\end{array}\right]$$

Subsequently, the inverse Clarke transformation is applied to reconstruct the three-phase currents from the α–β components.

10 $$\:\left[\begin{array}{c}{i}_{a}\\\:{i}_{b}\\\:{i}_{c}\end{array}\right]=\sqrt{\frac{2}{3}}\left[\begin{array}{cc}1&\:0\\\:\frac{-1}{2}&\:\frac{\sqrt{3}}{2}\\\:\frac{-1}{2}&\:\frac{-\sqrt{3}}{2}\end{array}\right]\left[\begin{array}{c}{i}_{\alpha\:}^{*}\\\:{i}_{\beta\:}^{*}\end{array}\right]$$

The source current consists of three components: the load current, the passive filter current, and the active compensating current that mitigates harmonics. Using these components, the peak source current (I peak (is determined, which is then employed to define the reference source current.

11 $$\:{I}_{source\_a(Ref.)}={I}_{Peak}\mathrm{sin}wt$$

12 $$\:{I}_{sourc{e}_{b\left(Ref.\right)}}={I}_{Peak}\mathrm{sin}(wt-120^\circ)$$

13 $$\:{I}_{source\_c(Ref.)}={I}_{Peak}\mathrm{sin}(wt+120^\circ)$$

The error is obtained from the difference between the reference source current and the actual source current. This error signal is then processed through the hysteresis current controller to generate the gate pulses for the inverter.

The third essential component of the Active Power Filter (APF) is the power inverter, which is responsible for generating the compensating current. This inverter utilizes MOSFET-based power electronic switches in conjunction with a DC-link capacitor to synthesize a current waveform that mirrors the amplitude of the undesired harmonic components but is phase-shifted by 180 degrees. By injecting this compensating current into the system, the harmonic components in the supply current are effectively mitigated, thereby enhancing the overall power quality and ensuring compliance with harmonic distortion standards.

The value of the reference voltage for the three-phase inverter can be expressed mathematically as follows^41^:$\:\:\:{V}_{dc\left(reference\right)}\ge\:\frac{2\times\:\sqrt{2}}{\sqrt{3}}\times\:{V}_{rms(L-L)}$

where Vdc is the DC-link voltage, V_rms(L−L) ​_ is the RMS value of the line-to-line AC voltage.

The operational steps of the proposed model are described as follows:

- Measure Voltages and Currents (Source Voltages, DC-Link Voltage, Load, Passive, Active and Source Currents).
- Apply the Clarke transformation to the load currents and Voltages.
- Calculate the power components (P, Q).
- Apply Hybrid Pelican- Grey Wolf Optimization.
- Generate Reference Current.
- Control Inverter Switching.
- Output Compensation Current.

## Optimization techniques

### Fractional order PI_λ_D_µ_ controller

FOPID controller is a generalized extension of the conventional PID controller, in which the integral and derivative components are defined with non-integer (fractional) orders rather than fixed integer values. This added degree of freedom allows for enhanced tuning flexibility, enabling more precise control over systems with nonlinear behavior and complex dynamic characteristics, where classical PID controllers may fall short^16^.

**Fig. 3:**
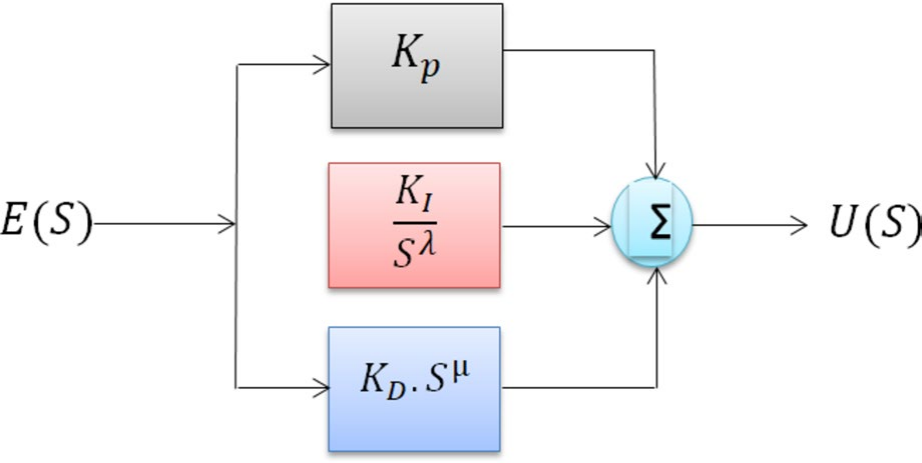
FOPID Controller.

The configuration of the FOPID controller is illustrated in Fig. 3, and its standard transfer function is expressed as:

14 $$\:{G}_{c}\left(S\right)=\frac{U\left(S\right)}{E\left(S\right)}={K}_{P}+\frac{{K}_{I}}{{S}^{\lambda\:}}+{K}_{D}.{S}^{\mu\:}$$

Where λ and µ are the integrating and derivative controller order respectively λ > 0, µ > 0, k_p_, k_i_, k_d_ are the controller parameters of FOPID.

One useful way to evaluate the dynamic performance of a CPID/FOPID control system is by looking at the integral error. When analyzing disturbance response or set-point changes, the integral error serves as the foundation for several commonly used performance criteria.

$$\:IAE={\int\:}_{0}^{\infty\:}\left|\mathrm{e}\right(\mathrm{t}\left)\right|\mathrm{d}\mathrm{t}ISE={\int\:}_{0}^{\infty\:}{\mathrm{e}\left(\mathrm{t}\right)}^{2}\mathrm{d}\mathrm{t}ISTE={\int\:}_{0}^{\infty\:}{\mathrm{t}}^{2}{\mathrm{e}\left(\mathrm{t}\right)}^{2}\mathrm{d}\mathrm{t}$$

$$\:ITAE={\int\:}_{0}^{\infty\:}\mathrm{t}\left|\mathrm{e}\right(\mathrm{t}\left)\right|\mathrm{d}\mathrm{t}ITSE={\int\:}_{0}^{\infty\:}\mathrm{t}{\mathrm{e}\left(\mathrm{t}\right)}^{2}\mathrm{d}\mathrm{t}$$

In this study the FOPID controller’s performance is evaluated using the Integral of Time and Absolute Error (ITAE), which serves as the loss function.

15 $$\:J={\int\:}_{0}^{{T}_{s}}\mathrm{t}\left|\mathrm{e}\right(\mathrm{t}\left)\right|\mathrm{d}\mathrm{t}$$

Where e (t) denotes the difference between the reference DC voltage and the actual DC voltage across the capacitor in the active filter circuit, T_s_ is simulation time.

The FOPID controller is implemented using the FOMCON Toolbox in Simulink, which handles the fractional order operators internally. The block uses an internal approximation method to realize the fractional derivatives and integrals, allowing the controller to operate directly in simulation without manually applying Oustaloup or CRONE techniques.

The fractional-order operators in the FOPID controller are approximated over a frequency range from 0.001 to 1000 rad/s, as set by the FOMCON Toolbox block. This range ensures that the system dynamics are accurately captured across the relevant operating conditions. The approximation of fractional-order operators in the FOPID controller uses a filter of order 5 which provides a balance between accuracy and computational efficiency. This choice is suitable for HAPF applications, as it allows reliable representation of system dynamics without imposing excessive computational burden during simulation.

Various optimization techniques have been employed to tune the parameters of the FOPID controller, with the primary objectives of accurately estimating the peak source current and maintaining the DC-link capacitor voltage at its desired reference level. The FOPID controller parameter limits were established through multiple experimental trials. Various values were tested to assess system behavior, and the limits were adjusted until stable and efficient performance was achieved. The tuning procedure began with K_p_ ​ to enhance the transient response, followed by K_i_ ​ to reduce the steady-state error, and finally K_d​_ to suppress oscillations while the fractional orders λ and µ were adjusted to further refine system dynamics. These experiments enabled the definition of practical and stable boundaries for the optimization process. All tuning and validation steps were conducted within the simulation environment to ensure consistent and reliable outcomes. The FOPID controller in the Simulink model is set with a 20µs sampling period, which is fast enough to accurately capture the behavior of HAPFs operating at 10–20 kHz. The POA–GWO optimization is performed offline to find the best controller parameters. Once these optimized values are obtained, they are applied in real time, ensuring reliable performance without violating the system’s high frequency constraints.

Effective voltage regulation is indicated by a minimal deviation between the actual and reference DC-link voltage, which directly correlates with improved system performance and stability. To achieve precise control and enhance overall performance, the FOPID controller parameters are optimally tuned using a hybrid Pelican–Grey Wolf Optimization (POA-GWO) algorithm. The idea of combining the Pelican Optimization Algorithm (POA) with the Grey Wolf Optimizer (GWO) stems from the complementary strengths of both methods. While GWO has already been successfully applied in similar power filter control applications due to its stable convergence and reliable local search ability, the POA offers stronger global exploration and adaptability. Merging their features allows the hybrid Pelican–GWO approach to balance exploration and exploitation more effectively, leading to faster convergence, improved tuning accuracy, and enhanced robustness of the FOPID controller in HAPFs. The transition from POA to GWO is done by passing the population of solutions obtained from POA directly to GWO as its initial population. Each solution keeps its position and fitness value, allowing GWO to refine the promising regions already identified. This sequential transfer effectively combines POA’s global exploration with GWO’s local exploitation, improving convergence and tuning reliability without additional modifications.

Optimization methods that rely on a single candidate solution, such as gradient-based or simplex-type techniques, are widely used due to their simplicity and relatively low computational burden. However, their performance is often influenced by the initial guess and the mathematical characteristics of the objective function. In nonlinear control problems with multiple interacting parameters, these methods may converge prematurely to suboptimal solutions.

In contrast, population-based algorithms evaluate and update several candidate solutions at the same time. This parallel search process increases diversity within the solution space and improves the ability to move away from local optima. As a result, these approaches are generally more reliable for complex tuning tasks.

In this work, tuning the FOPID controller for the HAPF system involves nonlinear dynamics, parameter coupling, and operating uncertainties. Under such conditions, a population-based strategy provides greater flexibility and robustness during the search process. While no optimization technique can be considered universally superior, the characteristics of the studied control problem make population-based algorithms a more suitable choice.

### Mathematical model

For the Active Filter, the dynamics of the inverter output current can be described by the series R-L branch as:

16 $$\:{v}_{s}-{v}_{inv.}=R.{i}_{f}+{L}_{f}.\frac{d{i}_{f}}{dt}$$

In the Laplace domain, this equation becomes:

17 $$\:{V}_{s}\left(S\right)-{V}_{inv}\left(S\right)=R{.I}_{f}\left(S\right)+{L}_{f}.S.{I}_{f}\left(S\right)$$

Hence, the inverter current can be expressed as:

18 $$\:{I}_{f}\left(S\right)=\frac{{V}_{s}\left(S\right)-{V}_{inv}\left(S\right)}{R+{L}_{f}.S}$$

For the DC link capacitor, the current flowing into the capacitor is related to the capacitor voltage derivative:

19 $$\:{i}_{dc}={C}_{dc}.\frac{d{v}_{dc}}{dt\:}$$

The corresponding DC-side power is given by:

20 $$\:{p}_{dc}={v}_{dc}.{i}_{dc}={{v}_{dc}.C}_{dc}.\frac{d{v}_{dc}}{dt\:}$$

At Steady State, the DC link voltage equals the reference value, $\:{v}_{dc}={Vdc}_{ref.}$and the DC-side power can be linearized as

21 $$\:{P}_{dc}={{V}_{d{c}_{ref}.}.C}_{dc}.S\:{V}_{dc}\left(S\right)\:$$

Finally, the small signal transfer function of the DC link voltage with respect to the DC power is:

22 $$\:\frac{{V}_{dc}\left(S\right)}{{P}_{dc}}=\frac{1}{{{V}_{d{c}_{ref}.}.C}_{dc}.S}$$

The DC link voltage dynamics are derived based on the power balance principle between the AC and DC sides of the converter. Assuming negligible converter losses, the instantaneous power equality can be expressed as:

$\:{P}_{ac}={P}_{dc}$, where the three phase AC-side power in the abc reference frame is given by.

For a balanced three phase system with sinusoidal voltages and currents, the total instantaneous becomes constant and can be written as

23 $$\:{P}_{ac}={\frac{3}{2}V}_{s0}\left(S\right).{I}_{f}\left(S\right)$$

where V_S0_​ represents the peak value of the phase voltage at the operating point.($\:{V}_{s0}=\frac{400\times\:\sqrt{2}}{\sqrt{3}}\:$).

Equating AC and DC powers yield

$$\:\frac{{V}_{dc}\left(S\right)}{{I}_{f}\left(S\right)}=\frac{\frac{3}{2}{V}_{s0}}{{{V}_{d{c}_{ref}.}.C}_{dc}.S}$$

By linearizing around the operating point (V_dc0_, Vs0) the small signal model of the DC-link voltage can be expressed as:

24 $$\:{G}_{P}\left(S\right)=\frac{{V}_{dc}\left(S\right)}{{I}_{f}\left(S\right)}=\frac{\frac{3}{2}{V}_{s0}}{{{V}_{d{c}_{ref}.}.C}_{dc}.S}$$

It should be noted that the dynamics of the AC-side filter (R_f_, Lf) are not included in this transfer function. This assumption is justified by the implementation of a fast inner current loop based on hysteresis current control with a narrow band of 0.01 A. Due to its high bandwidth, the current controller forces the filter current to track its reference almost instantaneously compared to the DC link voltage dynamics. Therefore, the inner current loop is approximated as unity gain, and only the DC link capacitor dynamics are considered in the outer voltage control design.

The FOPID controller regulates the DC link voltage to ensure active power balance, while harmonic extraction is achieved using instantaneous p–q theory.

The controller output represents the active power loss component, which is added to the harmonic power extracted from the instantaneous p–q theory to ensure power balance and maintain a constant DC-link voltage.

Substituting the controller given in (14) into the plant model, the open-loop transfer function becomes:

25 $$\:{G}_{OL}\left(S\right)={G}_{c}\left(S\right).{G}_{P}\left(S\right)={(K}_{P}+\frac{{K}_{I}}{{S}^{\lambda\:}}+{K}_{D}.{S}^{\mu\:})\times\:\frac{3}{2}.\frac{{V}_{s0}}{{{V}_{d{c}_{ref}.}.C}_{dc}.S}$$

and the corresponding closed loop transfer function is obtained as:

26 $$\:{G}_{CL}\left(S\right)=\frac{{G}_{c}\left(S\right).{G}_{P}\left(S\right)}{1+{G}_{c}\left(S\right).{G}_{P}\left(S\right)}=\frac{3}{2}.\frac{{{V}_{s0}(K}_{P}+\frac{{K}_{I}}{{S}^{\lambda\:}}+{K}_{D}.{S}^{\mu\:})}{{{V}_{d{c}_{ref}.}.C}_{dc}.S+\:{{V}_{s0}(K}_{P}+\frac{{K}_{I}}{{S}^{\lambda\:}}+{K}_{D}.{S}^{\mu\:})}$$

### Pelican optimization

The Pelican Optimization Algorithm (POA) is a recent nature-inspired metaheuristic that emulates the cooperative hunting strategies of pelicans. In this approach, each pelican is modeled as a search agent whose movements mimic the way pelicans locate and catch their prey. The algorithm alternates between exploration and exploitation to move closer to the optimal solution. Throughout the optimization process, pelicans adjust their positions by learning from the best solutions found so far, similar to how they adapt their hunting based on the surrounding environment and food sources. This mechanism helps the algorithm to search the solution space effectively while reducing the risk of being trapped in local optima. [47]

The Pelican Optimization Algorithm (POA) demonstrates significant flexibility and can be extended to tackle a variety of power quality issues, including voltage sag, swell, and unbalance, by tuning controllers in compensation devices such as DSTATCOM or UPQC. It is also effective for optimizing control parameters in renewable energy systems, enhancing converter efficiency, stabilizing voltage, and minimizing current distortion. In electric vehicle (EV) systems, POA can improve charging performance, power factor, and overall energy management. These applications underscore the algorithm’s adaptability across diverse power electronics environments, although practical implementation may encounter challenges related to computational time and real-time operation constraints^48–51^.

POA is particularly well suited for controlling HAPFs because it effectively handles the nonlinear and multi objective nature of FOPID controller tuning. By alternating between broad exploration of the solution space and focused local refinement, the algorithm avoids getting trapped in suboptimal solutions and ensures stable performance. This makes POA robust for improving harmonic suppression, maintaining voltage stability, and achieving reliable power quality under varying load conditions.

**Fig. 4:**
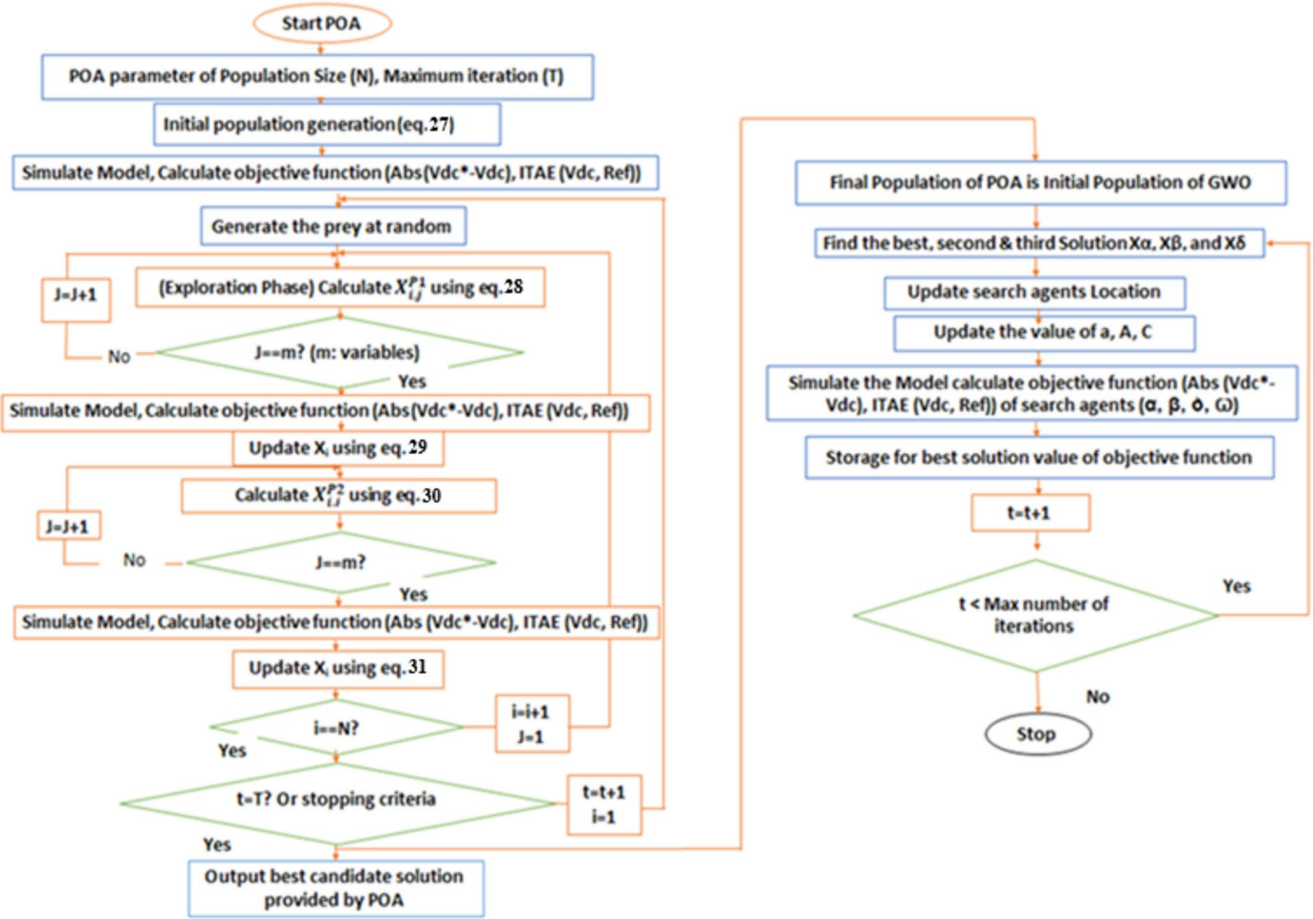
Hybrid Pelican-Grey Wolf Optimization Flow Chart.

Figure 4 presents hybrid Pelican-Grey Wolf Optimization flow chart; the equations in the charts are as follows:

The equations of the Pelican Optimization Algorithm (POA) presented in this section are adopted from [47].

27 $$\:{X}_{i,j}={l}_{j}+rand.\left({u}_{j}-{l}_{j}\right)\:,\:\:\:\mathrm{i}=1,\:2,\dots\:,\mathrm{N},\:\:\:\:\mathrm{j}=\mathrm{1,2},\dots\:,\mathrm{m}$$

where X_i, j_ is the value of the j^th^ variables, N is the number of population members (search agents=4), m is the number of problem variables (for PID m=3), rand is a random number in interval[0,1], *l*_j_ is the lower bound & *u*_*j*_ is the upper bound.

28 $$\:{X}_{i,j}^{P1}=\left\{\begin{array}{c}{X}_{i,j}+rand.\left({p}_{j}-I.{X}_{i,j}\right)\:\:\:,\:{F}_{p}<{F}_{i}\\\:{X}_{i,j}+rand.\left({X}_{i,j}-{p}_{j}\right)\:\:\:\:,\:else,\:\:\:\:\end{array}\right.$$

(28)$\:{\mathrm{w}\mathrm{h}\mathrm{e}\mathrm{r}\mathrm{e}\:X}_{i,j}^{P1}$ is the new status of the j^th^ pelican in the j^th^ dimension based on phase 1, *I* Random number which is equal to one or two, *p*_*j*_ is the location of prey in the j^th^ dimension, *F*_*p*_ is the Objective Function value of the$\:\:\:{p}_{j}$

29 $$\:{X}_{i}=\left\{\begin{array}{c}{X}_{i}^{P1},\:{F}_{i}^{P1}<{F}_{i}\:\:\:\:\:\:\:\:\:\:\:\:\:\:\:\:\\\:{X}_{i}+rand.\left({X}_{i,j}-{p}_{j}\right),\:\:else,\:\:\:\:\end{array}\right.$$

$\:{\mathrm{W}\mathrm{h}\mathrm{e}\mathrm{r}\mathrm{e}\:X}_{i}^{P1}$is the new status of the *i*^*th*^ pelican,$\:{F}_{i}^{P1}\:$is the objective Function value of the $\:{X}_{i}^{P1}$ based on Phase1

30 $$\:{X}_{i,j}^{P2}={X}_{i,j}+R.\left(1-\frac{t}{T}\right).\left(2.rand-1\right).{X}_{i,j}$$

Where $\:{X}_{i,j}^{P2}$is the new status of the jth pelican in the jth dimension based on phase 2, R is a constant=0.2, tis iteration counter, T is the maximum number of iterations.

31 $$\:{X}_{i}=\left\{\begin{array}{c}{\mathrm{X}}_{\mathrm{i}}^{\mathrm{P}2},\:{\mathrm{F}}_{\mathrm{i}}^{\mathrm{P}2}<{\mathrm{F}}_{\mathrm{i}}\:\:\:\:\:\:\:\:\:\:\:\:\:\:\:\:\\\:{\mathrm{X}}_{\mathrm{i}},\:\:\:\:\:\:\:else,\:\:\:\:\:\:\:\:\:\:\:\:\:\:\:\:\:\:\:\end{array}\right.$$

Where $\:{X}_{i}^{P2}$ is the new status of the i_th_ pelican, $\:{F}_{i}^{P2}$ is the objective function value of the status $\:{X}_{i}^{P2}$based on Phase2.

After reaching to maximum iterations the final values will be initial values to Grey Wolf Optimization.

### Grey wolf optimization

The Grey Wolf Optimization (GWO) algorithm is inspired by the social hierarchy and group hunting strategy of grey wolves. In this method, the optimal solution is represented as the prey, while the wolves simulate the pack moving toward it^52^.

The GWO algorithm is known for its strong global search capability, fast convergence, and ability to avoid local minima, making it effective for accurately tuning FOPID controllers in hybrid active power filter applications. For these reasons, GWO was chosen to be applied following the Pelican algorithm to enhance optimization performance.

The update of their positions is expressed through mathematical equations that describe how the wolves encircle and approach the prey. The mathematical formulation of the Grey Wolf Optimizer (GWO) employed in this work is adopted from the original model presented in^52^.

32 $$\:\overrightarrow{D}=\left|\overrightarrow{C\:}.\:\overrightarrow{{X}_{p}}\left(t\right)-\overrightarrow{X\left(t\right)}\right|$$

33 $$\:\overrightarrow{X}\left(t+1\right)=\overrightarrow{{X}_{p}}\left(t\right)-\overrightarrow{A}.\overrightarrow{D}$$

Where t is the current iteration, $\:\overrightarrow{A}\:and\:\overrightarrow{C}$ are the coefficient vectors, $\:\overrightarrow{{X}_{p}}$ is the prey position vector, $\:\overrightarrow{X}$ is a grey wolf position vector

34 $$\:\overrightarrow{A}=2.\overrightarrow{a}.\overrightarrow{r1}.\overrightarrow{a}$$

35 $$\:\overrightarrow{C}=2.\overrightarrow{r2}$$

r1, r2 are random vectors in the interval between 0 and 1.

To simulate the hunting strategy of grey wolves mathematically, the best three solutions are considered as alpha, beta, and delta, since they are assumed to have better knowledge about the prey’s location. These top solutions are preserved during the process, while the remaining search agents, including the omegas, update their positions based on the guidance of the leading wolves. the following formulas are

36 $$\:\overrightarrow{{D}_{\alpha\:}}=\left|\overrightarrow{{C}_{1}\:}.\:\overrightarrow{{X}_{\alpha\:}}\left(t\right)-\overrightarrow{X\left(t\right)}\right|,\:\overrightarrow{{D}_{\beta\:}}=\left|{\overrightarrow{C\:}}_{2}.\:\overrightarrow{{X}_{\beta\:}}\left(t\right)-\overrightarrow{X\left(t\right)},\:\overrightarrow{{D}_{\delta\:}}=\left|\overrightarrow{{C}_{3}\:}.\:\overrightarrow{{X}_{\delta\:}}\left(t\right)-\overrightarrow{X\left(t\right)}\right|\right|$$

$$\:\overrightarrow{{X}_{1}}=\overrightarrow{{X}_{\alpha\:}}-\overrightarrow{{A}_{1}}.\left(\overrightarrow{{D}_{\alpha\:}}\right),\:\overrightarrow{{X}_{2}}=\overrightarrow{{X}_{\beta\:}}-\overrightarrow{{A}_{2}}.\left(\overrightarrow{{D}_{\beta\:}}\right),\:\overrightarrow{{X}_{3}}=\overrightarrow{{X}_{\delta\:}}-\overrightarrow{{A}_{3}}.\left(\overrightarrow{{D}_{\delta\:}}\right)$$

$\:\overrightarrow{{D}_{\alpha\:}}$,$\:\:\overrightarrow{{D}_{\beta\:}}$, $\:\overrightarrow{{D}_{\delta\:}}$ is the distance between prey and alpha wolf, beta wolf and delta wolf respectively.

$\:\overrightarrow{{X}_{1}}$,$\:\:\overrightarrow{{X}_{2}}$,$\:\:\overrightarrow{{X}_{3}}$ is the prey position according to alpha wolf, beta wolf and delta wolf respectively.

37 $$\:\overrightarrow{X}\left(t+1\right)=\frac{\overrightarrow{{X}_{1}}+\overrightarrow{{X}_{2}}+\overrightarrow{{X}_{3}}}{3}$$

Each iteration of POA, GWO, or the hybrid POA–GWO requires evaluating the objective function for all search agents. For every agent, the FOPID controller parameters (Kp, Ki, Kd, α, β) are applied in the Simulink model, and the resulting system response is used to calculate the performance. This process can be computationally intensive due to the number of agents and iterations, but it is performed offline. Once the optimal parameters are determined, the FOPID controller can operate in real-time without any delay, ensuring stable and reliable performance.In both POA and GWO, the population size remains constant throughout the optimization process. In the hybrid POA–GWO approach, the same population obtained from POA is directly transferred to GWO without any change in size, ensuring consistency in solution refinement.

## Results and analysis

### Passive filter

**Fig. 5:**
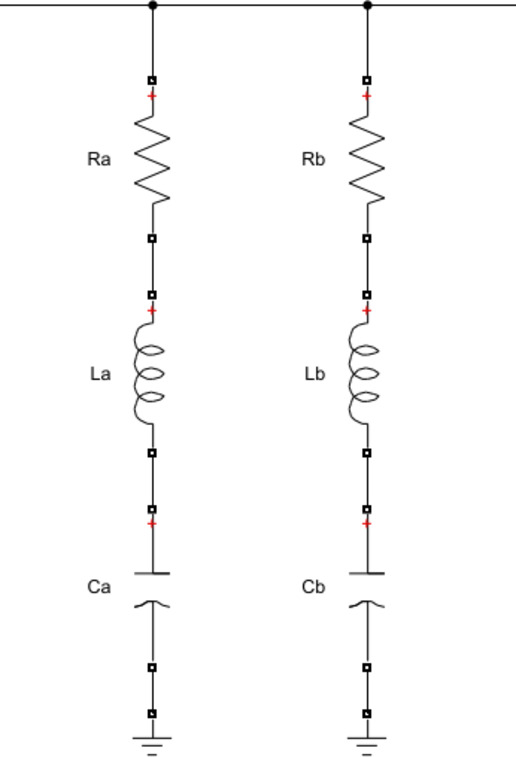
Configuration of two single-tuned passive filters designed for the selected harmonic orders.

**Fig. 6:**
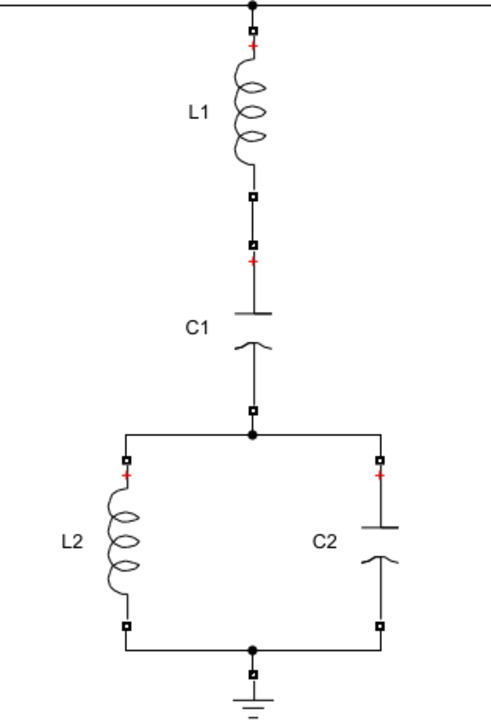
Equivalent circuit of the double-tuned passive filter for the same harmonic orders.

Double-Tuned Passive Filters (DTPFs) were strategically implemented to suppress specific harmonic orders that are commonly generated by nonlinear loads. In this study, two DTPFs were designed: the first aimed at attenuating the 5th and 7th harmonic components, and the second focused on mitigating the 11th and 13th harmonics. The filter design process was conducted using the Equivalent Circuit Method (ECM), a well-established technique in harmonic filter design as described in the reference^53^. These filters were connected in parallel with the voltage source between the source and the load to provide low impedance paths for the targeted harmonic frequencies, thereby significantly reducing their presence in the supply current. The performance of the DTPFs was evaluated through simulation using MATLAB/Simulink. Figures 5 and 6 show block diagram of two single tuned passive filters for the selected harmonic orders and equivalent circuit of the double tuned passive filter for the same harmonic orders.

The parameters of the single-tuned passive filter are first calculated, and then the equivalent circuit method is applied to obtain the parameters of the double-tuned passive filter. First, the values of the single passive filter parameters were obtained using the following equations^54^.

- Determine the capacitor power capacity Qc which equal to the load reactive power.
- Determine the capacitance of the capacitor from the following equation.

38 $$\:C=\frac{{Q}_{c}}{2\pi\:fm{V}^{2}}$$

where f is the power frequency, m is branches number for all phases and V is the phase voltage.

- Determine the inductance of the inductor from the equation.

39 $$\:L=\frac{1}{4{\pi\:}^{2}{f}^{2}{h}^{2}C}$$

where h is the order of harmonic.

- Determine the resistance used in the filter.

40 $$\:R=\frac{2\pi\:hfL}{{Q}_{n}}$$

where $\:{Q}_{n}$ is the quality factor which has been assigned a value of 50 in this study.

Second, to design of Double tuned shunt passive filter the equivalent circuit method in reference 31 is used. The traditional double filtered filter has a series of resonant and shunt resonance circuits. The series circuit gives series resonant frequency (ωs) and parallel circuit gives parallel resonant frequency (ωp). These two resonant frequencies enable the filtering of two dominant low-order current harmonics from the power system using a single circuit.

The following final equations are used in double tuned design:

41 $$\:{C}_{1}={C}_{a}+{C}_{b}$$

42 $$\:{L}_{1}=\frac{1}{{C}_{a}{\omega\:}_{a}^{2}+{C}_{b}{\omega\:}_{b}^{2}}$$

43 $$\:{L}_{2}=\frac{\left(1-\frac{{\omega\:}_{a}^{2}}{{\omega\:}_{s}^{2}}\right)\left(1-\frac{{\omega\:}_{a}^{2}}{{\omega\:}_{p}^{2}}\right)}{{C}_{1}{\omega\:}_{a}^{2}}$$

44 $$\:{C}_{2}=\frac{1}{{L}_{2}{\omega\:}_{p}^{2}}$$

The transfer function of the passive filter is given by the following Equation:

45 $$\:\frac{{I}_{pf}\left(s\right)}{{V}_{s}\left(S\right)}=\frac{1}{\left[S.{L}_{1}+\frac{1}{S.{C}_{1}}+\frac{\frac{S}{{C}_{2}}}{\left[{S}^{2}+\frac{1}{{C}_{2}{L}_{2}}\right]}\right]}$$

where Ipf denotes the phase current of the passive filter flowing through the series combination of L_1_ and C_1_, and Vs represents the corresponding phase supply voltage.

**Fig. 7:**
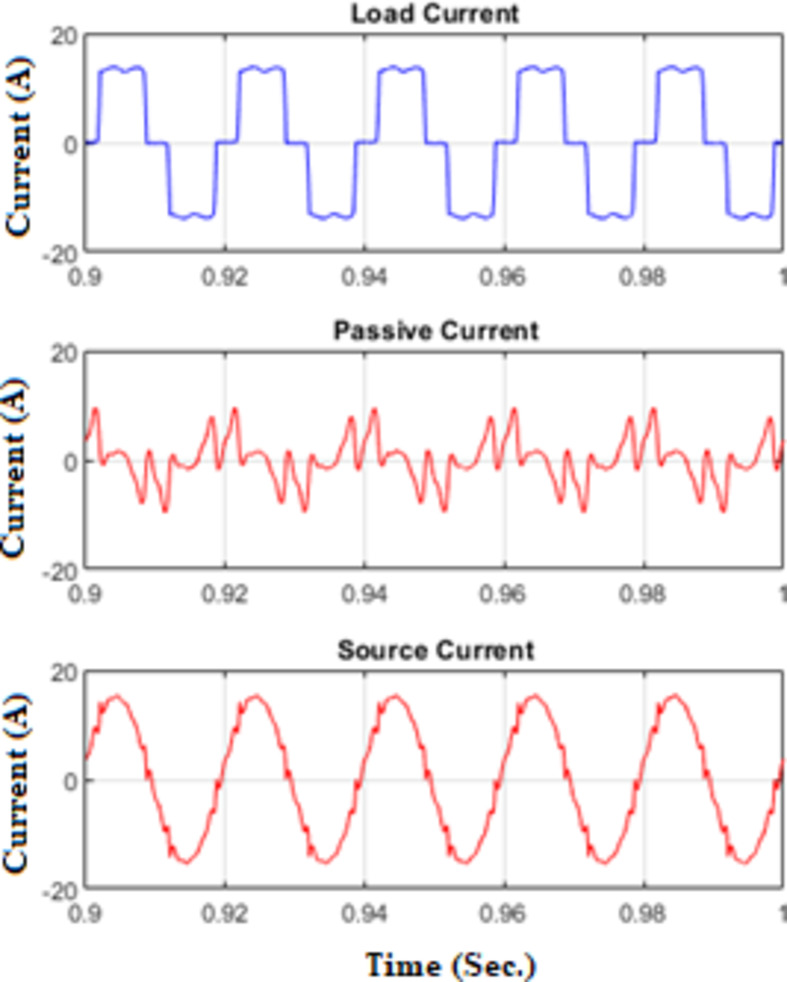
Waveforms of load, passive filter, and source currents for phase a under passive filter only connected.

**Fig. 8:**
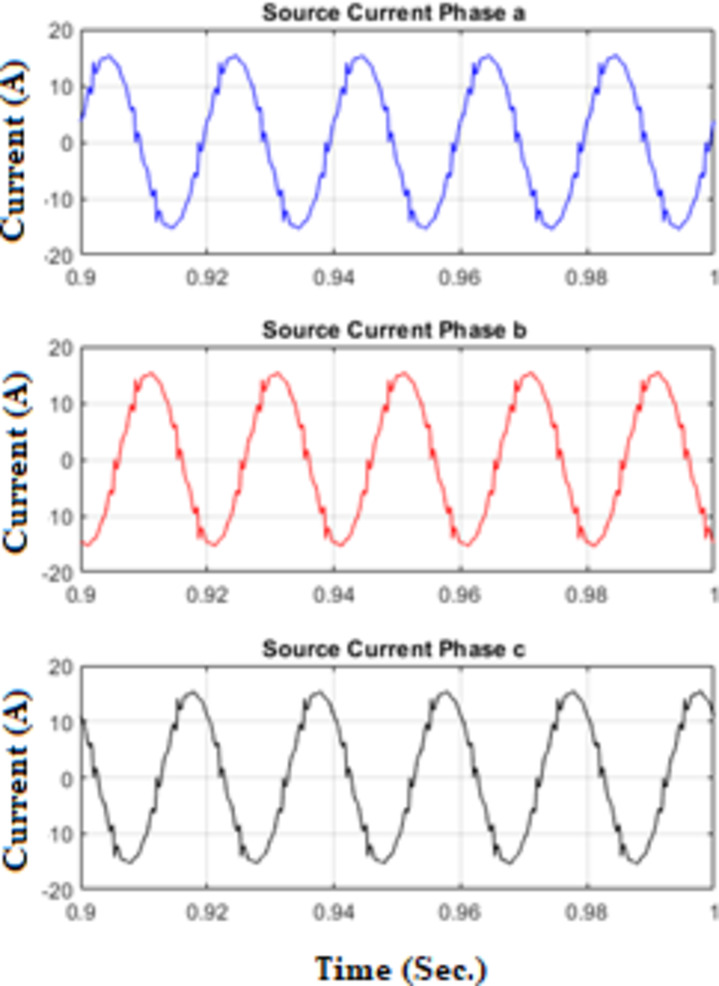
Source currents (phases a, b, and c) under passive filter only connected.

**Fig. 9:**
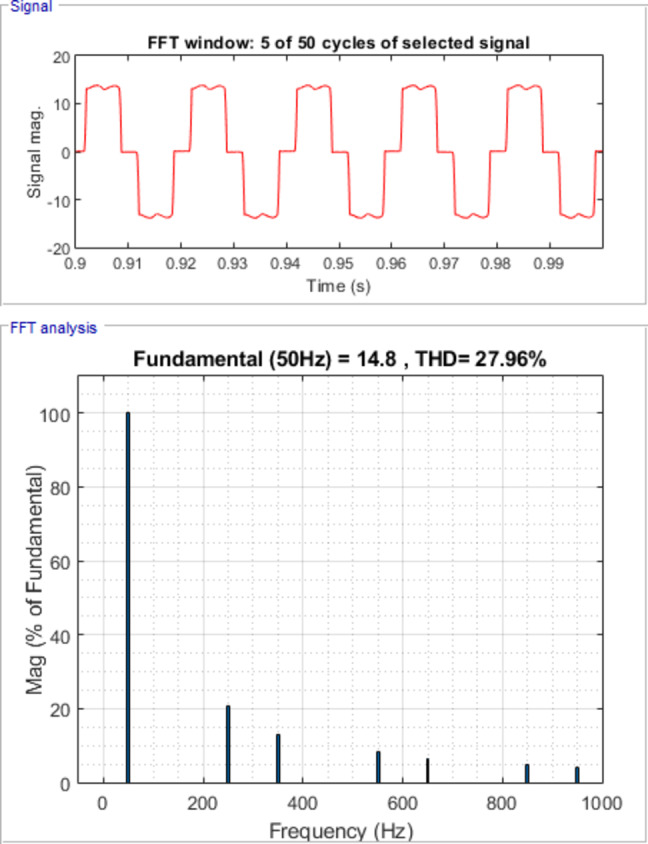
Harmonic Spectrum of Load Current.

**Fig. 10:**
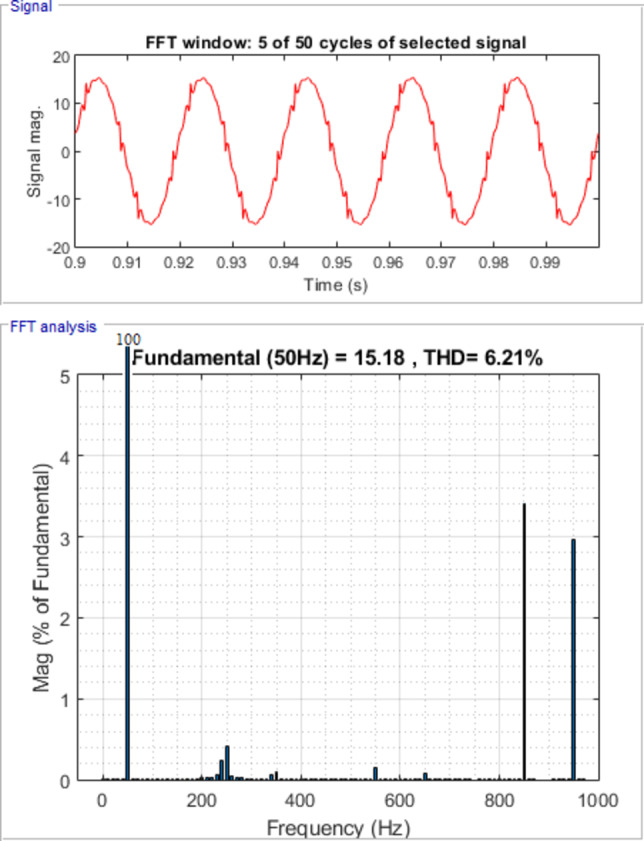
Source Current Waveform and its harmonic spectrum for phase a under passive filter only connected.

Figure 7 shows waveforms of load, passive filter, and source currents for phase a. Figure 8 presents the resulting three-phase source current waveforms, which appear more sinusoidal after filtering. To further validate the filtering performance, Figs. 9 and 10 display the harmonic spectrum of the load current and the source current, respectively. The effectiveness of the DTPFs in improving power quality is quantitatively confirmed by Table 1, which shows a notable reduction in Total Harmonic Distortion (THD). Specifically, the THD of the source current was reduced from 27.96% (pre-filtering) to 6.21% (post-filtering). This substantial improvement demonstrates the DTPFs’ capability in effectively suppressing dominant low-order harmonics and enhancing overall system performance and compliance with power quality standards.

**Table 1:** Load and Source Currents values and their THD for phase a, b &c after passive filter installation.

	Load Current		Source Current	
	I_fund. (A)	THD (%)	I_fund. (A)	THD (%)
Phase a	14.8	27.96	15.18	6.21
Phase b	14.8	27.96	15.18	6.21
Phase c	14.8	27.96	15.18	6.21

### . Hybrid filter optimized by hybrid (POA-GWO)

In this section, the performance of the Hybrid Active Power Filter optimized using the hybrid Pelican- Grey Wolf Optimization hybrid (PO-GWO) technique is presented.

The better performance of the Pelican–GWO sequence is due to each algorithm being used where it is most effective. POA first explores the search space widely to find promising solutions and avoid getting stuck in local minima, while GWO then fine-tunes these solutions with focused local search. This order provides more stable convergence and more reliable FOPID controller tuning than using the reverse sequence or standalone algorithms.

**Fig. 11:**
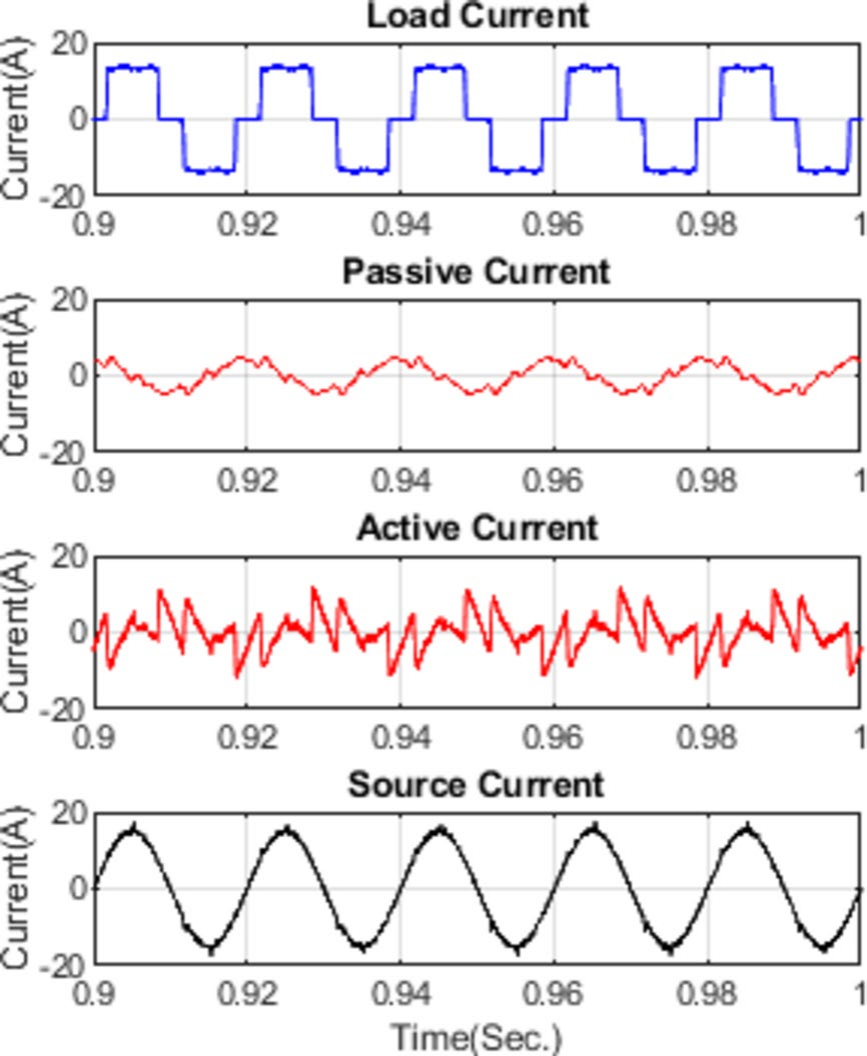
Various waveforms of Hybrid Filter for FOPID using Hybrid Pelican-Grey Wolf optimization.

**Fig. 12:**
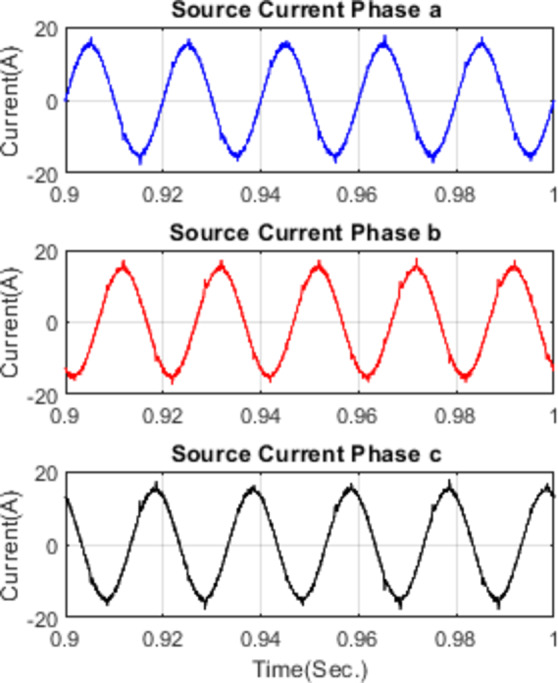
Source Currents for phase a, b and c under FOPID using Hybrid Pelican-Grey Wolf optimization.

**Fig. 13:**
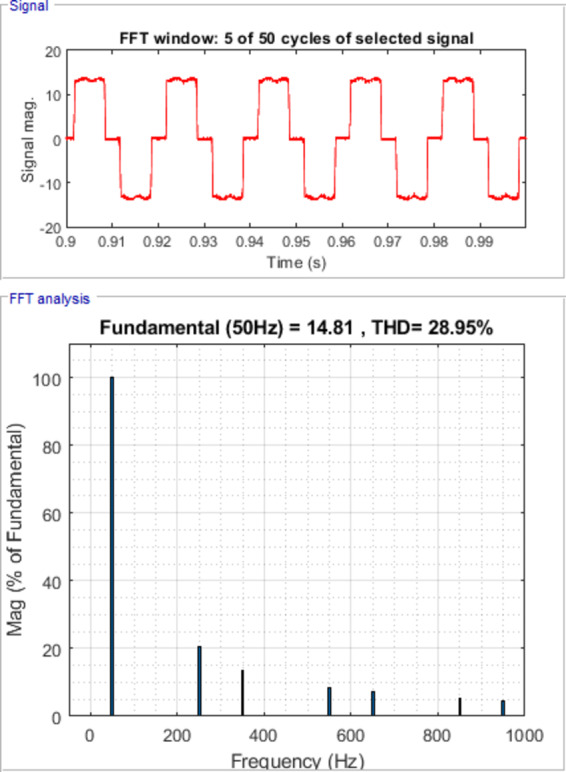
Harmonic Spectrum of Load Current.

**Fig. 14:**
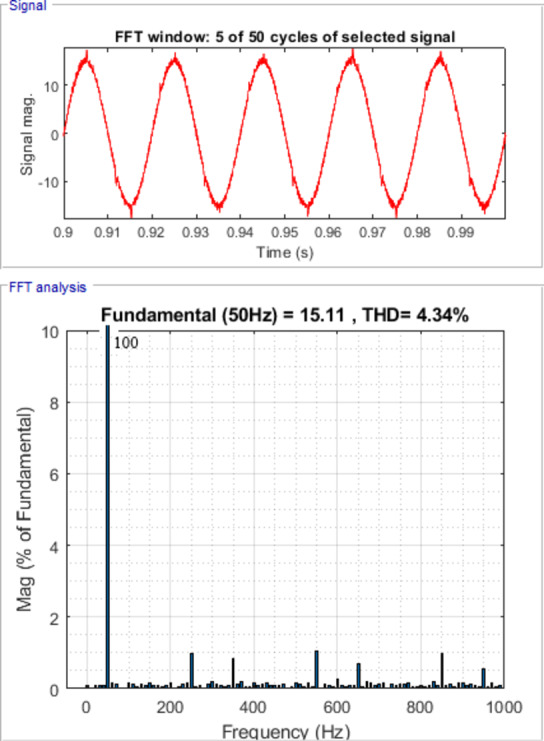
Harmonic Spectrum of Source Current under FOPID using Hybrid Pelican-Grey Wolf optimization.

- Figure 11 illustrates the waveforms of the load current, passive current, active current, and source current when the FOPID parameters are tuned using hybrid (PO-GWO). The optimization technique effectively minimizes the Total Harmonic Distortion (THD) in the system, ensuring improved performance of the hybrid filter.
- Figure 12 displays the three-phase waveforms of the resulting source current, highlighting the successful harmonic compensation by the hybrid filter under the influence of hybrid (PO-GWO) optimization.
- Figs. 13 and 14 present the harmonic spectrum for both the load current and the source current, respectively. The results demonstrate a significant reduction in harmonic content in the source current, with the hybrid (PO-GWO) optimized filter outperforming traditional methods in terms of THD reduction.

The figures clearly illustrate the effectiveness of the hybrid filter with PO-GWO, highlighting how the FOPID tuning improves power quality and enhances harmonic compensation. Table 2 demonstrates the improvement in power quality by showing the reduction in THD values. For example, the distortion level decreased from 28.95% to 4.34% for phase a, which clearly highlights the effectiveness of the proposed filtering and control approach in minimizing harmonics.

Robust control strategies, such as Sliding Mode Control (SMC) and H-infinity (H∞) control, are widely recognized for their ability to handle disturbances and uncertainties. However, these methods often face practical challenges SMC can introduce high frequency chattering in the control signal, which may be undesirable in power electronic systems, while H∞ control usually requires an accurate mathematical model and can be computationally demanding^55^^,^^57^.

The proposed Hybrid Pelican–GWO FOPID controller offers a practical alternative that does not rely on precise system modeling. It maintains robustness against parameter variations and external disturbances while remaining computationally efficient and straightforward to implement. This combination of performance and practicality makes it particularly suitable for applications such as the HAPF system, where a balance between control effectiveness and implementation feasibility is critical^57^.

While a direct comparison with SMC or H∞ controllers is not included in this study, the discussion highlights the advantages of the proposed approach and provides a clear justification for its use in the considered system.

**Table 2:** Load and Source Currents values and their THD for phase a, b &c after hybrid filter installation under FOPIDC.

	Load Current		Source Current	
	I_fund. (A)	THD (%)	I_fund. (A)	THD (%)
Phase a	14.81	28.95	15.11 A	4.34
Phase b	14.8	29.01	15.13	4.58
Phase c	14.76 A,	29.17	15.13 A,	4.57

**Fig. 15:**
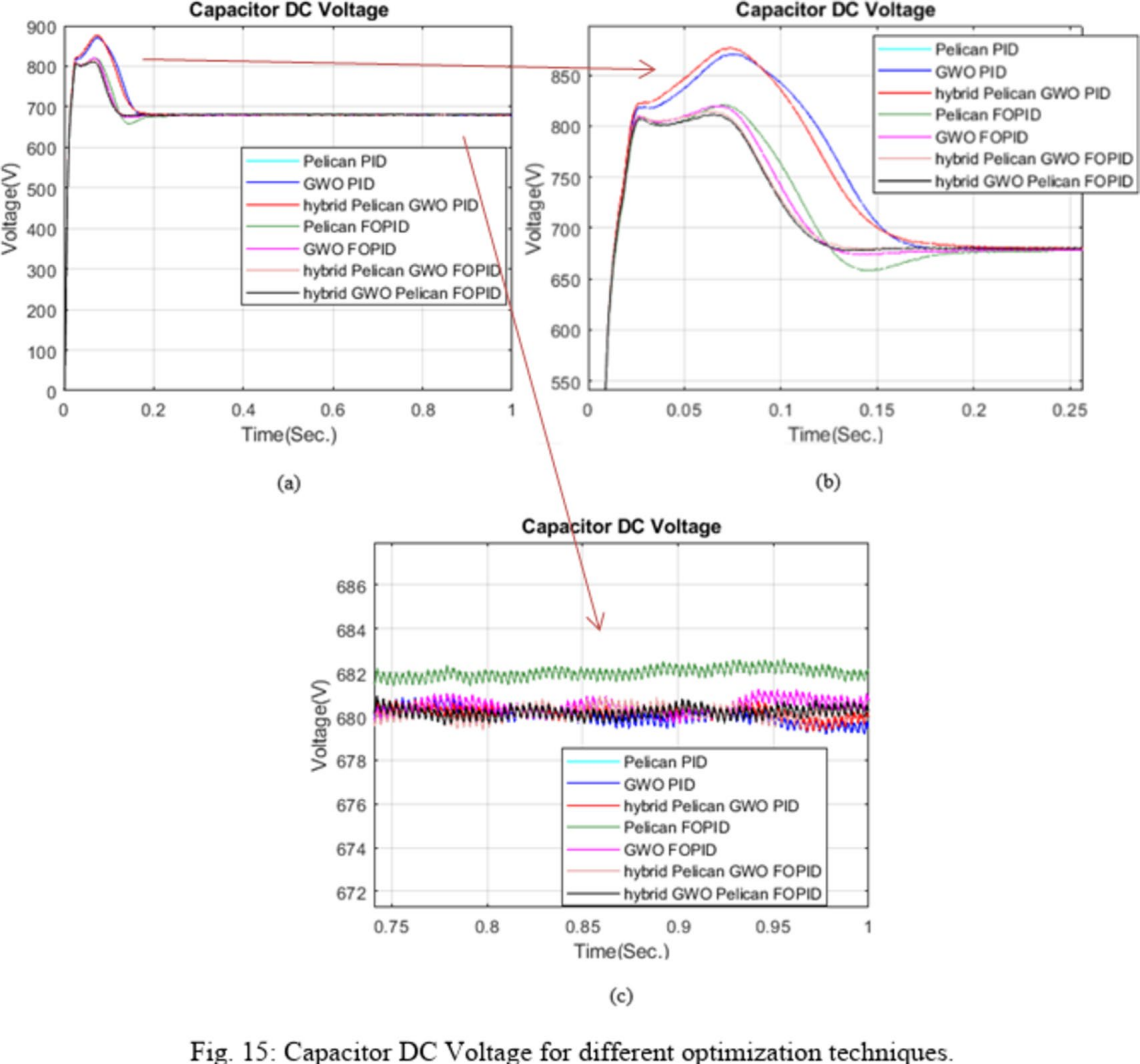
Capacitor DC Voltage for different optimization techniques.

Figure 15 clearly demonstrates the superior dynamic performance of the FOPID controller compared to the conventional PID controller. The FOPID controller achieved reduced overshoot and a shorter settling time, reflecting its improved control efficiency. Moreover, this enhanced transient behavior implies improved stability margins, as the controller exhibits better damping characteristics and faster recovery from disturbances compared to PID-based schemes optimized using other methods.

Table 3 presents a comparative evaluation of the dynamic performance of the different optimized controllers for DC link voltage regulation. It is evident that the fractional-order controllers (FOPID-based approaches) outperform the conventional PID-based methods in terms of reduced overshoot and faster settling time. Among all tested algorithms, the Hybrid GWO Pelican FOPID Hybrid Filter achieves the lowest overshoot (19.2568%) and one of the fastest settling times (0.1139 s), indicating superior transient performance. Additionally, the steady state error for all controllers remains negligible, confirming accurate DC-link voltage tracking. Settling time is computed within ± 2% tolerance band. Compared to PID-based configurations, the hybrid FOPID approaches demonstrate improved damping characteristics and enhanced transient stability. These results verify that the proposed hybrid optimization strategy significantly enhances the dynamic regulation capability of the DC-link voltage.

**Table 3:** Dynamic performance comparison of the optimized for DC-link voltage regulation.

Optimization Algorithm with Controller Type	Overshoot (%)	Stead-State (%)	Settling Time (Sec.)	ITAE (0–1 s)
POA-PIDC, Hybrid Filter	28.9047	−0.006	0.1566	1.515
GWO-PIDC Hybrid Filter	28.0341	0.0283	0.1563	1.592
Hybrid POA-GWO-PIDC Hybrid Filter	28.9047	−0.006	0.1566	1.515
POA-FOPIDC, Hybrid Filter	20.6936	−0.3133	0.1650	1.682
GWO-FOPIDC Hybrid Filter	20.5557	−0.0814	0.1170	0.927
Hybrid POA-GWO-FOPIDC Hybrid Filter	19.7364	−0.0199	0.1167	0.740
Hybrid GWO-Pelican FOPID Hybrid Filter	19.2568	−0.0376	0.1139	0.748

### Result under unbalanced Condition

**Fig. 16:**
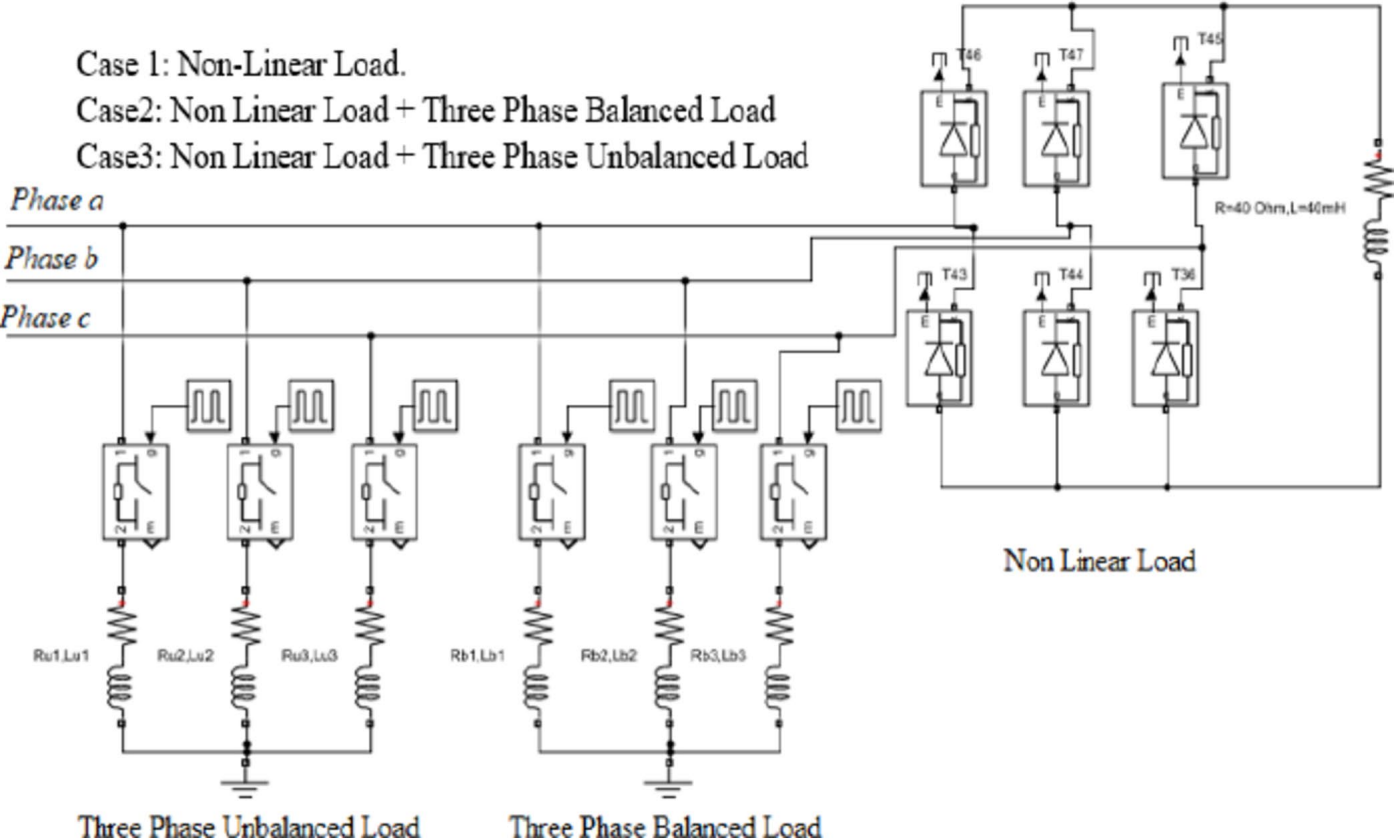
Simulation of the Load Cases.

To evaluate the performance of the Hybrid Active Power Filter (HAPF) under unbalanced load conditions, simulations were conducted for three distinct scenarios, as illustrated in Fig. 16:

1. Case 1: A nonlinear load (three-phase uncontrolled bridge rectifier with RL load) is connected from t = 0 to t = 0.5 seconds.

2. Case 2: Three phase balanced RL load (40 Ω resistor with 40 mH inductor ,40 Ω resistor with 40 mH inductor and 40 Ω resistor with 40 mH inductor) are connected to Phase A, Phase B and Phase C, in parallel with the nonlinear load, from t = 0.5 to t = 0.7 seconds.

3. Case 3: Three phase unbalanced RL load (32 Ω resistor with 34 mH inductor ,40 Ω resistor with 40 mH inductor and 48 Ω resistor with 47 mH inductor) are connected respectively to Phase A, Phase B and Phase C, in parallel with the nonlinear load, from t=0.7 to t = 1 seconds.

**Fig. 17:**
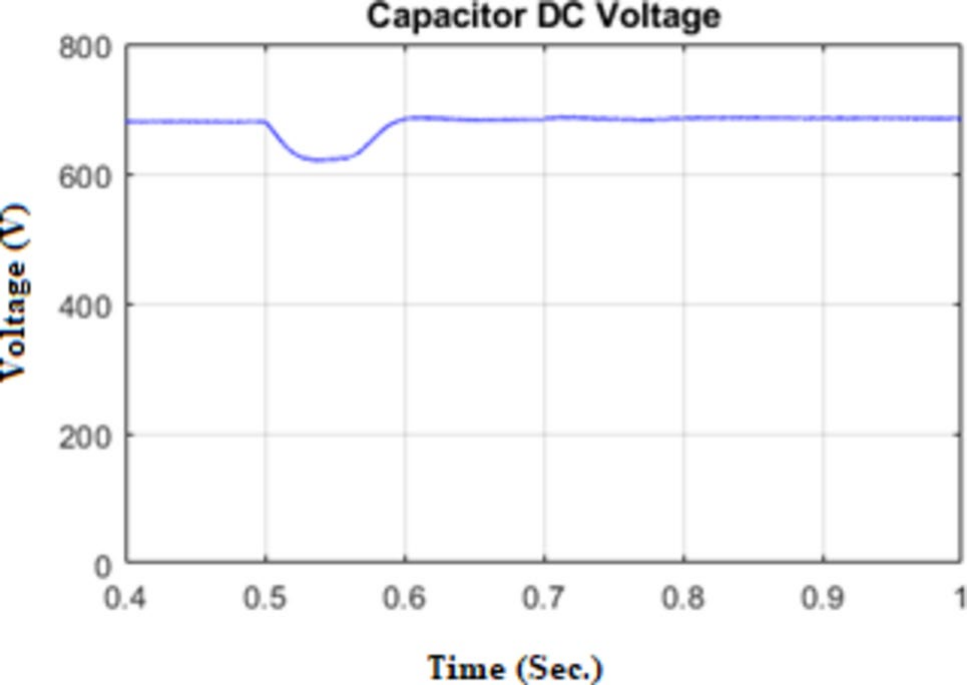
Capacitor DC Voltage under unbalanced Load.

Figure 17 shows the DC-link capacitor voltage response across the three loading conditions, providing a clear indication of its behavior in response to dynamic load variations.

**Fig. 18:**
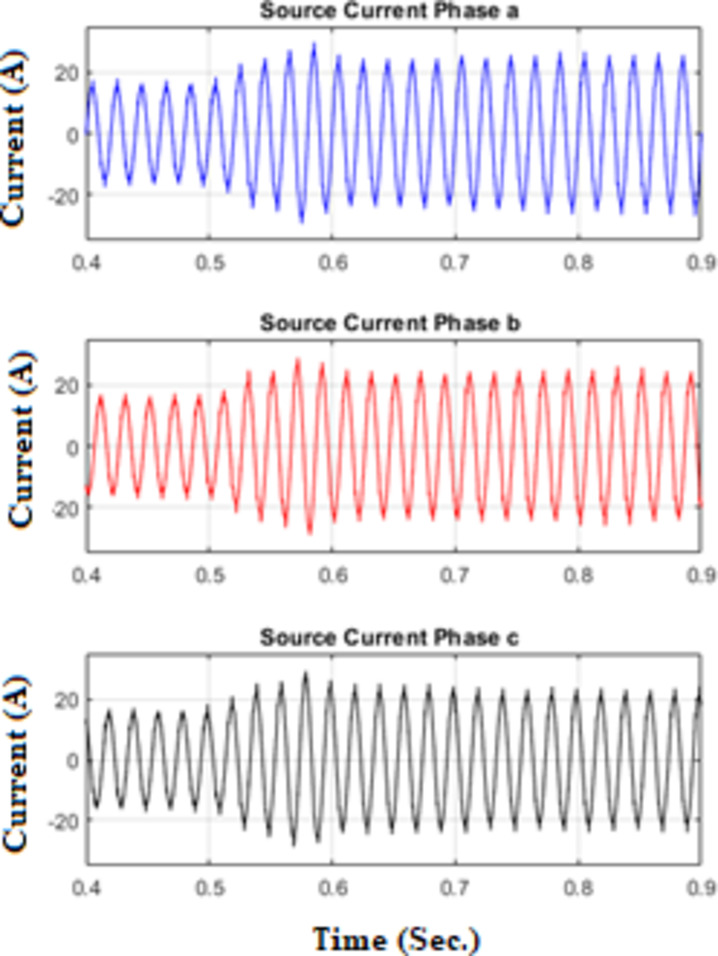
Sources Currents under unbalanced condition.

**Fig. 19:**
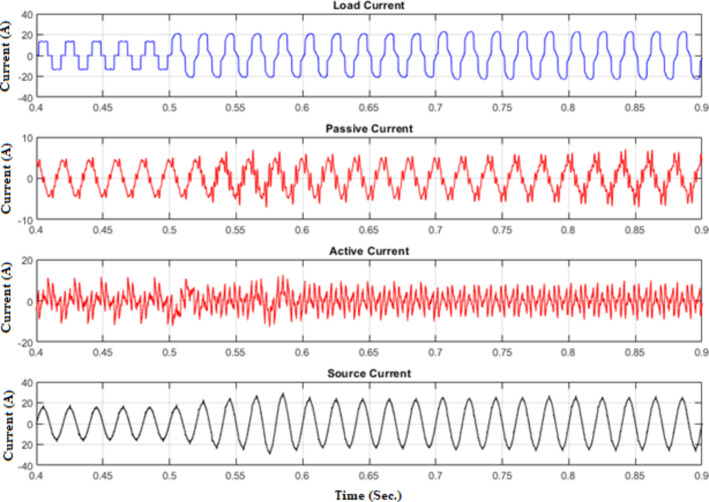
Various Waveforms of Hybrid Filter for FOPID using Pelican-Grey Wolf optimization Under unbalanced Load conditions for phase a.

Figure 18 shows three phase source currents under unbalanced condition, and Fig. 19 illustrates the waveforms of the load current, passive filter current, active filter current, and source current under unbalanced conditions. The FOPID controller parameters were tuned using the hybrid Pelican Grey Wolf Optimization (POA-GWO) algorithm.

The results clearly demonstrate the HAPF’s ability to compensate for unbalanced loads, effectively maintaining a stable, balanced, and harmonic-free source current, even under dynamically changing load conditions.

### Comparing between optimization techniques

Table 3 presents the parameter limits and final optimized values for both PID and FOPID controllers. It also includes the Integral of Time-weighted Absolute Error (ITAE) results up to the simulation time for various optimization methods, the voltage deviation, ($\:{\varDelta\:\boldsymbol{V}}_{\boldsymbol{d}\boldsymbol{c}}$) which is defined as the difference between the DC-link reference voltage (Vdc_ref = 680 V) and the average measured DC-link voltage (Vdc_actual). The results indicate that the hybrid optimization-based FOPID controller (POA–GWO) outperforms the other approaches in terms of control accuracy and dynamic performance. Additionally, the Table 4 highlights the deviation of the capacitor voltage from its reference value under each control strategy.

The optimized FOPID parameters λ and µ were constrained within the range of 0 to 1. The obtained values (λ = 0.344, 0.841, 0.966, 0.962; µ = 0.281, 0.157, 0.791, 0.141) are all within these physically meaningful limits, ensuring stable and robust controller operation for the HAPF system.

**Table 4:** Parameters values for different optimization techniques.

Optimization Algorithm with Controller Type	$\:{\boldsymbol{K}}_{\boldsymbol{P}}$	$\:{\boldsymbol{K}}_{\boldsymbol{I}}$	$\:{\boldsymbol{K}}_{\boldsymbol{D}}$	λ	µ	$\:{\varDelta\:\boldsymbol{V}}_{\boldsymbol{d}\boldsymbol{c}}\left(\mathbf{V}\right)$ At 1 s.
Parameters Limits for PID Controller	(15–100)	(0.1–20.1)	(0.01–0.1.01.1)			
Parameters Limits for FOPID Controller	(15–100)	(5–50)	(0.01–0.1.01.1)	(0–1)	(0–1)	
POA-PIDC, Hybrid Filter	37.899	20	0.098	--	--	0.165
GWO-PIDC Hybrid Filter	42.094	20	0.029	--	--	0.474
Hybrid POA-GWO-PIDC Hybrid Filter	37.934	19.565	0.097	--	--	0.165
POA-FOPIDC, Hybrid Filter	34.436	50	0.019	0.344	0.281	−1.923
GWO-FOPIDC Hybrid Filter	41.791	50	0.062	0.841	0.157	−0.595
Hybrid POA-GWO-FOPIDC Hybrid Filter	39.347	50	0.063	0.966	0.791	−0.253
Hybrid GWO-Pelican FOPID Hybrid Filter	41.566	50	0.057	0.962	0.141	−0.324

For the FOPID controller, the hybridization process was applied in two sequences.

- In the first case by using hybrid Pelican–Grey Wolf optimization, the final values obtained from the Pelican Optimization Algorithm were used as initial values for the Grey Wolf Optimizer.
- In the second case by using hybrid Grey Wolf- Pelican optimization the sequence was reversed.

The results reveal that both hybrid configurations produced similar ITAE and capacitor voltage deviation (ΔV) values and exhibited nearly identical dynamic behavior.

Pelican–GWO algorithm FOPID controller. Specifically, the ITAE achieved using the Pelican algorithm alone is 1.682, which decreases to 0.927 when employing the Grey Wolf Optimizer. However, the hybrid Pelican–GWO approach attains the lowest ITAE value of 0.740, confirming its enhanced optimization capability and improved control performance compared to the individual algorithms. Moreover, the hybrid GWO–Pelican configuration achieved an ITAE value of 0.748, which is very close to that of the hybrid Pelican–GWO, indicating that both hybridization directions provide strong optimization potential, with the Pelican–GWO showing a slight advantage in performance.

**Fig. 20:**
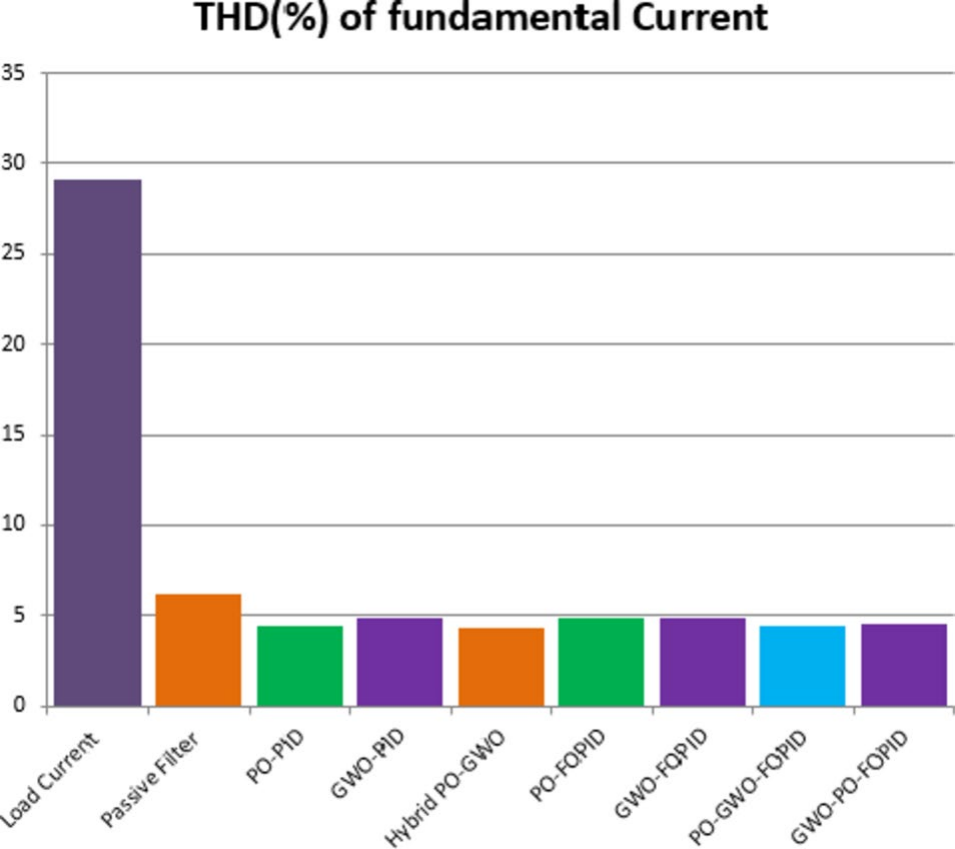
Comparative analysis of THD (%) for all cases.

The Total Harmonic Distortion (THD) results further confirm the effectiveness of the proposed hybrid approach using FOPID controller. The THD is 4.83% when using the Pelican algorithm and slightly higher at 4.89% with the GWO algorithm. In contrast, the hybrid Pelican-GWO achieves the lowest THD value of 4.42%, indicating its superior ability to enhance power quality and minimize harmonic distortion compared to the individual optimization techniques. Therefore, all the obtained results are within the acceptable range specified by the standard IEEE Std. 519–2014^58^. Fig. 20 shows Comparative analysis of THD (%) for the load at case 1.

Table 5 reviews THD of Load and Source Currents Values of phase a for different optimization techniques.

**Table 5:** THD of Load and Source Currents Values of (phase a) for different optimization techniques.

Optimization Technique	Time	Load Current (Phase a)		Source Current (Phase a)	
		I_fund. (A)	THD (%)	I_fund. (A)	THD (%)
PO- PID	From t=0 s.to t=0.5 s.	14.78	29.05	15.12	4.44
	From t=0.5 s. up to 0.7 s.	22.28	19.31	22.54	3.47
	From t=0.7 s. up to 1 s.	24.35	17.37	23.73	3.81
GWO-PID	From t=0 s.to t=0.5 s.	14.79	29.11	15.16	4.92
	From t=0.5 s. up to 0.7 s.	22.29	19.27	22.45	3.45
	From t=0.7 s. up to 1 s.	24.34	17.46	23.74	3.73
Hybrid PO-GWO	From t=0 s.to t=0.5 s.	14.8	29	15.11	4.30
	From t=0.5 s. up to 0.7 s.	22.31	19.28	22.53	3.36
	From t=0.7 s. up to 1 s.	24.33	17.41	23.77	4.09
PO-FOPID	From t=0 s.to t=0.5 s.	14.82	28.97	15.15	4.83
	From t=0.5 s. up to 0.7 s.	22.3	19.21	22.18	3.91
	From t=0.7 s. up to 1 s.	24.32	17.55	23.74	4.12
GWO-FOPID	From t=0 s.to t=0.5 s.	14.76	29.24	15.17	4.89
	From t=0.5 s. up to 0.7 s.	22.33	19.12	22.34	3.50
	From t=0.7 s. up to 1 s.	24.34	17.44	23.69	3.50
PO-GWO-FOPID	From t=0 s.to t=0.5 s.	14.83	28.90	15.14	4.42
	From t=0.5 s. up to 0.7 s.	22.33	19.10	22.43	3.29
	From t=0.7 s. up to 1 s.	24.35	17.39	23.76	4.01
GWO-PO-FOPID	From t=0 s.to t=0.5 s.	14.8	29.02	15.13	4.58
	From t=0.5 s. up to 0.7 s.	22.3	19.20	22.38	3.13
	From t=0.7 s. up to t=1 s.	24.36	17.38	23.72	3.52

### Computational parameters

To ensure a fair and consistent comparison among all implemented optimization algorithms, identical computational settings were adopted for each case study. For all optimization techniques, the population size was set to 4, and the maximum number of iterations was fixed at 200. The lower and upper bounds of the controller parameters were presented in Table 4.

The objective function used for parameter tuning was the ITAE performance index, defined over a simulation time of one second with a sampling time of 20 micro second.

All simulations were performed in MATLAB/Simulink environment (version 2018a) on a system equipped with AMD Ryzen 3 PRO 3300U w processor and 16 GB RAM to guarantee reproducibility of results.

Table 6 presents the simulation time and the computational (algorithm execution) time for each investigated case study, including Pelican-PID, GWO-PID, Hybrid Pelican-GWO PID, Pelican-FOPID, GWO-FOPID, Hybrid Pelican-GWO FOPID, and Hybrid GWO-Pelican FOPID.

The execution time of each optimization algorithm was calculated using MATLAB’s built in tic–toc functions, which measure the elapsed time between the start and end of the algorithm implementation. The total number of objective function evaluations is equal to Search agents × No. of Itererations (800), where each evaluation requires a detailed switching simulation in Simulink. To evaluate the computational performance, two different times were considered: the algorithm execution time per iteration and the Simulink simulation time per search agent. The algorithm execution time measures how long the MATLAB optimization routine takes to update all search agents in one iteration, while the simulation time shows how long Simulink needs to simulate the system for a single candidate over a 1-second period. The observed simulation times are expected for power system simulations, as they include the detailed switching behavior of the inverter and associated power electronics.

The results show that hybrid algorithms, such as POA–GWO and GWO–Pelican, take slightly longer per iteration compared to standalone POA or GWO. This is expected, since combining two optimization methods adds some extra computation. However, all algorithms used the same number of iterations and the same population size, so the comparison is fair.

Looking at the simulation times, hybrid controllers often complete the 1-second Simulink simulation as fast or faster than the standalone algorithms. This suggests that the parameters found by the hybrid methods improve system dynamics and make the numerical simulation more stable.

**Table 6:** Simulink Simulation Time and Algorithm Execution Time for All Implemented Controllers.

Optimization Algorithm with Controller Type	algorithm execution time/Iteration (average of 10 runs) (Sec.)	Total Time= Iterations × Search Agents × algorithm execution time/Iteration (Sec.)	Simulation Time /Search agent (t=1 Sec.)
POA-PIDC, Hybrid Filter	Phase1:0.0573, Phase2:0.0090	53.04	44.4567 Sec.
GWO-PIDC Hybrid Filter	0.0754	60.32	41.1063 Sec.
Hybrid POA-GWO-PIDC Hybrid Filter	0.1418	113.44	40.7152 Sec.
POA-FOPIDC, Hybrid Filter	Phase1:0.0551, Phase2:0.0122	53.84	46.9685 Sec.
GWO-FOPIDC Hybrid Filter	0.0695	55.6	50.88669 Sec.
Hybrid POA-GWO-FOPIDC Hybrid Filter	0.1369	109.52	45.2150 Sec.
Hybrid GWO-Pelican FOPID Hybrid Filter	0.1369	109.52	46.8087 Sec.

### Statistical Analysis

Due to the extremely high computational cost of the detailed switching model, statistical consistency of each algorithm was assessed based on intra run population variance, mean, and convergence behavior, using the iteration wise fitness data recorded in Excel.

Table 7 presents the statistical evaluation of the ITAE values obtained over 200 iterations for each optimization algorithm. The mean value reflects the overall performance level, while the standard deviation and variance indicate the convergence stability throughout the optimization process. Lower dispersion values imply more consistent search behavior and reduced oscillations during convergence. the Hybrid GWO-Pelican FOPID Hybrid Filter demonstrates the lowest mean ITAE value with minimal standard deviation and variance, indicating superior convergence stability and consistent optimization performance compared to the other algorithms.

**Table 7:** Overall statistical performance of the optimization algorithms over 200 iterations in terms of ITAE.

Optimization Algorithm with Controller Type	Mean	Standard Deviation	Variance	Best	Worst
POA-PIDC, Hybrid Filter	2.013	0.924	0.849	1.515	5.312
GWO-PIDC Hybrid Filter	1.865	0.654	0.426	1.592	5.652
Hybrid POA-GWO-PIDC Hybrid Filter	1.515	0.000	0.000	1.515	1.515
POA-FOPIDC, Hybrid Filter	2.145	0.979	0.954	1.682	6.799
GWO-FOPIDC Hybrid Filter	1.181	0.638	0.405	0.927	5.491
Hybrid POA-GWO-FOPIDC Hybrid Filter	0.822	0.230	0.0525	0.740	1.532
Hybrid GWO-Pelican FOPID Hybrid Filter	0.758	0.025	0.0006	0.748	0.927

### Stability Analysis

**Fig. 21:**
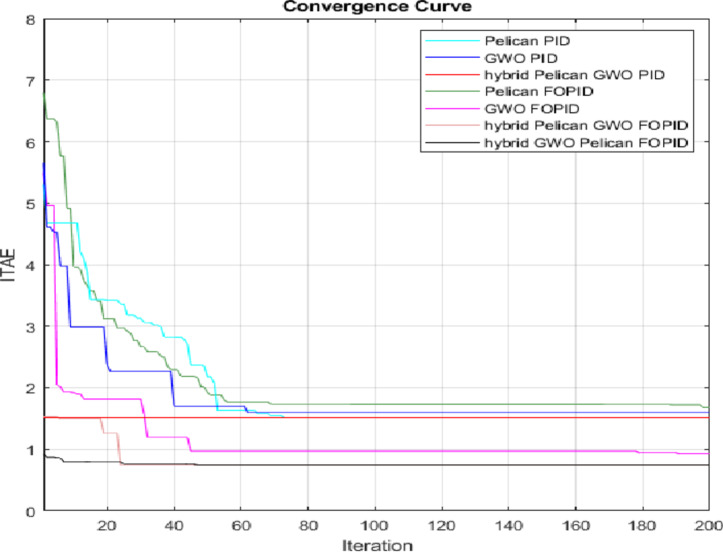
Convergence Curves for all Optimization Techniques.

The convergence curves in Fig. 21 highlights how the different optimization algorithms perform. It shows that the hybrid FOPID controller converges more quickly and achieves a lower objective function value than both the individual algorithms and the traditional controllers. This means the hybrid approach is more effective at reaching the optimal solution, as it needs less iteration while still delivering higher accuracy. For a clearer comparison, the convergence characteristics of the algorithms were analyzed.

The Pelican Optimization based FOPID controller achieved an objective value of 1.738 after 69 iterations, while the GWO reached a better value of 0.9698 within 45 iterations. Notably, the hybrid Pelican-GWO algorithm attained the lowest objective value of 0.7399 in only 24 iterations and the hybrid GWO-Pelican optimization achieved an objective value of 0.7677 after 25 iterations, which is very close to that of the hybrid Pelican-GWO. This demonstrates that the hybrid optimization approach not only provides superior optimization accuracy but also achieves a significantly faster convergence rate, making it a more efficient and practical choice for real-time and hardware-oriented applications.

### Sensitivity Analysis

**Fig. 22:**
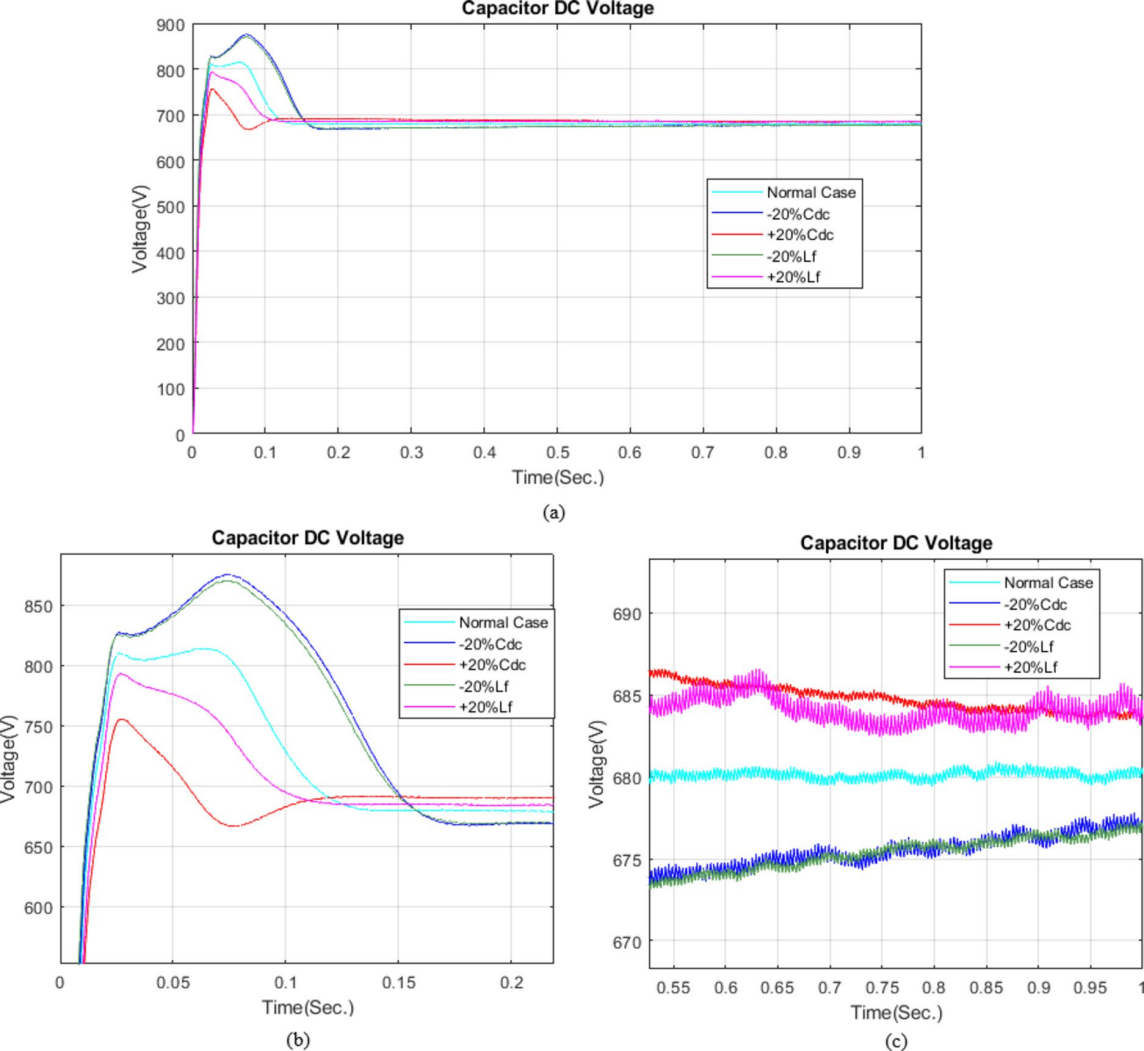
DC-link voltage response under ± 20% parameter variations.

For the Hybrid Pelican GWO optimized FOPID controller, Fig. 22 illustrates the DC link voltage response under ± 20% variations in the DC-link capacitor (Cdc) and filter inductance (Lf). It can be observed that parameter variations mainly affect the transient behavior, resulting in different overshoot levels and settling times. Specifically, reducing Cdc or Lf by 20% increases the overshoot (up to 28.72% for − 20% Cdc and 27.99% for − 20% Lf) and prolongs the settling time (up to 0.1501 s and 0.1490 s, respectively), while increasing these parameters by 20% reduces overshoot (down to 13.96% for + 20% Cdc and 16.63% for + 20% Lf) and shortens the settling time (to 0.0857 s and 0.1012 s, respectively), as summarized in Table 8. Despite these variations, all cases converge to the reference DC link voltage of 680 V with negligible steady-state error (ranging from − 0.52% to 0.54%). No instability or divergence is observed throughout the simulation period, confirming that the proposed control strategy ensures stable operation and robust DC-link voltage regulation under significant parameter uncertainties.

**Table 8:** Effect of Parameter Variations on System Dynamic Response.

Parameter Variation Case	Overshoot (%)	Stead-State (%)	Settling Time (Sec.)
Normal	19.7364	−0.0199	0.1167
−20%Cdc	28.7213	0.4512	0.1501
+20%Cdc	13.9586	−0.3923	0.0857
−20%Lf	27.9976	0.5401	0.1490
+20%Lf	16.6287	−0.5189	0.1012

### Open loop and closed loop system analysis

**Fig. 23:**
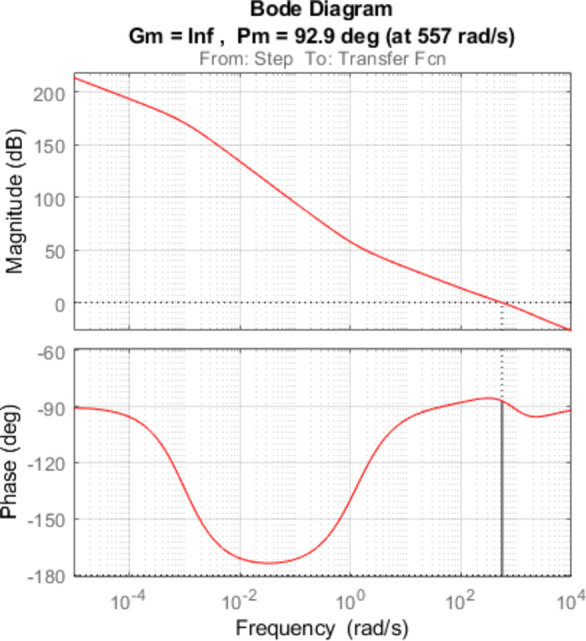
Open-loop Bode plot of the system.

From Fig. 23, the Bode plot of the open-loop system, given by Eq. (25), indicates a gain crossover frequency of approximately 557 rad/s (≈ 88.7 Hz) and a phase margin of 92.9°, reflecting very strong stability. The gain margin is infinite, meaning the system does not reach a phase crossover at 0 dB, which further demonstrates its robustness.

The magnitude plot shows a high low-frequency gain, ensuring good tracking, and it gradually decreases, crossing 0 dB at the expected frequency. The phase plot drops to around − 170° at low frequencies but recovers near the crossover, resulting in a high phase margin. This large phase margin corresponds to an overdamped response with minimal overshoot and very smooth behavior, indicating that the system is both robust and reliable. The visual impression of a steep descent is typical in FOPID-controlled systems due to the fractional-order dynamics, but it does not imply reduced stability.

The closed-loop step response demonstrates a minimal overshoot of approximately 1.14% and an essentially zero steady-state error (−0.0024%). The system settles almost instantaneously, indicating a fast and stable closed-loop operation. These results confirm that the proposed FOPID-controlled HAPF maintains robust tracking performance and effectively suppresses disturbances, which is consistent with the high phase margin observed in the open-loop Bode plot.

Overall, the analysis confirms that the system is stable and conservative. The high phase margin contributes to minimal overshoot and excellent disturbance rejection, making the design well-suited for sensitive power control applications.

### Real time validation

Real-time validation is currently under development as part of the ongoing experimental implementation. Due to the extensive calibration and safety procedures inherent to power electronic systems, the experimental results are not yet finalized and will be reported in a forthcoming study.

## Conclusion

This study introduced an optimized control approach to improve the performance of Hybrid Active Power Filters (HAPFs) for power quality enhancement. The proposed method relies on a hybrid optimization framework that combines the Pelican Optimization Algorithm (POA) with the Grey Wolf Optimizer (GWO) to tune the parameters of a Fractional Order PID (FOPID) controller. Simulation results showed a clear improvement in harmonic mitigation, with the Total Harmonic Distortion (THD) reduced from 28.95% to 4.34%.

Beyond harmonic reduction, the optimized controller demonstrated better dynamic behavior, including faster settling time, lower overshoot, and stable operation under different load conditions. It was also observed that applying POA first, followed by GWO, provided slightly better performance than the reverse sequence, as this arrangement allows broad exploration of the search space before fine adjustment of the controller parameters. In addition, combining the HAPF with a double-tuned passive filter helped maintain nearly sinusoidal source currents, highlighting the practical benefit of integrating active and passive filtering.

In general, the results confirm that the proposed hybrid optimization strategy is an effective solution for improving power quality in systems with nonlinear loads. Future work will focus on experimental validation through real-time implementation, as well as extending the approach to systems with grid disturbances and renewable energy integration, to further assess its applicability in practical power system environments.

## Data Availability

The datasets used and/or analyzed during the current study are available from the corresponding author on reasonable request.
